# Positive affect, surprise, and fatigue are correlates of network flexibility

**DOI:** 10.1038/s41598-017-00425-z

**Published:** 2017-03-31

**Authors:** Richard F. Betzel, Theodore D. Satterthwaite, Joshua I. Gold, Danielle S. Bassett

**Affiliations:** 1 Department of Bioengineering, University of Pennsylvania, Philadelphia, PA 19104 USA; 2 Neuropsychiatry Section, Department of Psychiatry, University of Pennsylvania, Philadelphia, PA 19104 USA; 3 Department of Neuroscience, University of Pennsylvania, Philadelphia, PA 19104 USA; 4 Department of Electrical and Systems Engineering, University of Pennsylvania, Philadelphia, PA 19104 USA

## Abstract

Advances in neuroimaging have made it possible to reconstruct functional networks from the activity patterns of brain regions distributed across the cerebral cortex. Recent work has shown that flexible reconfiguration of human brain networks over short timescales supports cognitive flexibility and learning. However, modulating network flexibility to enhance learning requires an understanding of an as-yet unknown relationship between flexibility and brain state. Here, we investigate the relationship between network flexibility and affect, leveraging an unprecedented longitudinal data set. We demonstrate that indices associated with positive mood and surprise are both associated with network flexibility – positive mood portends a more flexible brain while increased levels of surprise portend a less flexible brain. In both cases, these relationships are driven predominantly by a subset of brain regions comprising the somatomotor system. Our results simultaneously suggest a network-level mechanism underlying learning deficits in mood disorders as well as a potential target – altering an individual’s mood or task novelty – to improve learning.

## Introduction

The human brain is a complex network composed of neural elements and their interconnections with one another^1, 2^. One approach for interrogating the brain’s network organization is via the so-called resting state fMRI paradigm, wherein blood oxygen level dependent (BOLD) signals are recorded from subjects in the absence of any explicit task instructions. The functional connectivity (FC) between pairs of brain regions is estimated as the statistical interdependency – e.g. temporal correlation, coherence, mutual information, *etc*. – of spontaneous fluctuations in their BOLD time series, and can be interpreted as a measure of communication between brain regions^3, 4^. FC networks can be constructed by calculating FC for all pairs of brain regions. The result is a square matrix whose elements represent the magnitude of FC between each pair of regions.

Recent work has shown that FC network organization fluctuates over timescales of seconds to minutes^5–7^, opening the possibility of studying the time-varying properties of FC networks by tracking the instantaneous communication patterns among brain regions^8–11^. One means of characterizing dynamic FC networks is by their community structure^12–14^, which refers to decompositions of a network into densely-interconnected sub-networks or “communities”^15, 16^. In the context of FC networks, communities represent collections of brain regions that tend to preferentially connect to (communicate with) one another, while weakly connecting to the rest of the brain^17–19^. The community structure of dynamic networks, then, tracks the ongoing formation and dissolution of communities over time^20, 21^. Such a measure makes it possible to identify the brain’s temporal core and periphery of flexible brain regions that tend to change their community assignment over time versus inflexible ones that maintain a consistent assignment^22^.

Intriguingly, network flexibility has been shown to correlate with both learning rate and cognitive flexibility^12, 23^. These abilities are not static but can vary considerably over time and as a function of an individual’s affective state. For example, learning often shows an “inverted-U” relationship with arousal, with optimal learning at moderate levels of arousal^24^. Together, these findings imply that the influence of affective state on learning and cognition may involve modulations of brain network flexibility. However, virtually nothing is known about such modulations.

A potential simple and intuitive affect-related driver of daily variations in brain network flexibility is mood^25^. Mood can fluctuate normally over time scales ranging from minutes to weeks. Moreover, mood can influence learning, for example by biasing the perception of reward outcomes^26^. These biases are thought to arise from neurophysiological changes in neurotransmitter systems linked to arousal^27^. A second potential driver of fluctuations in network flexibility is surprise, which refers to acute mismatches between expectation and reality^28^. Surprise plays a key role in memory formation^29^, associative learning^30^, error correction^31^, and the allocation of attentional resources^32^. However, the network-level mechanisms of these processes in the human brain remain largely unknown.

We hypothesized that different affective components would be associated with enhanced brain network flexibility, potentially explaining the observations that more flexible brains display greater cognitive flexibility and better learning^12, 23^ and may be related to attentional state^33^. To address this hypothesis, we leveraged data from the *MyConnectome Project*, which included extensive longitudinal neuroimaging and behavioral data from a single subject acquired over a period of approximately one year^34, 35^. We extracted two affect-related indices from the behavioral data – one tracked the subject’s mood while the other tracked surprise – and showed that these indices were correlated with global network flexibility, a relationship that was driven primarily by the flexibility of the somatomotor network. These results simultaneously suggest a network-level mechanism underlying learning deficits in mood disorders as well as a potential target – altering an individual’s mood or state of surprise – to improve learning.

## Results

To address the hypothesis that network flexibility is associated with mood, we analyzed data collected as part of the *MyConnectome Project*, which includes extensive longitudinal neuroimaging and behavioral data from a single participant acquired over a period of approximately one year^34, 35^. As part of the imaging protocol, the participant underwent multiple resting state functional magnetic resonance imaging (fMRI) scans. Using a novel, participant-specific parcellation, the cerebral cortex was divided into 630 non-overlapping regions. Each of these regions was also assigned to a cognitive system, explicitly representing a category of cognitive function (Fig. S1).

**Figure 1:**
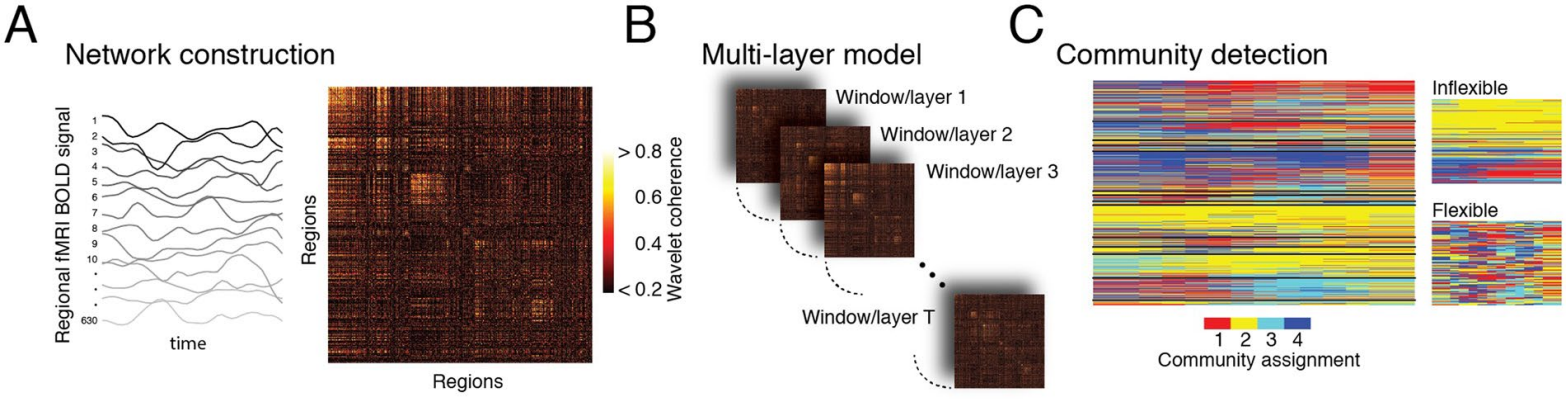
Schematic figure illustrating network construction, multi-layer network model, and community detection output. (**A**) Functional connectivity networks were generated by calculating the magnitude squared wavelet coherence for all pairs of regional fMRI BOLD time series^73^. The resulting coherence estimates are arranged in a region-by-region functional connectivity matrix. (**B**) Functional connectivity matrices for non-overlapping windows were arranged sequentially to form a multi-layer network – each layer corresponds to a window of time during the fMRI scan session^12^. For the purpose of multi-layer community detection, each brain region was coupled to itself across sequential windows. (**C**) Typical output of the multi-layer community detection algorithm^20^. Each network node (brain region) is assigned to a community in each layer. Here we show an example where four communities were detected. The consistency of those communities across adjacent layers determines a region’s flexibility^12^. The sub-panels show the 10% least flexible (top) and most flexible (bottom) brain regions.

Using these data, we estimated network flexibility for each region and on average across the brain. This entailed first dividing regional fMRI BOLD time series into non-overlapping windows. Within each window, we estimated functional connectivity between all pairs of brain regions using a magnitude-squared wavelet coherence^12^. The result was an ordered set of functional connectivity matrices, each of which represented a layer in a multi-layer network representation^21^. Functional connectivity measures the strength of a statistical relationship between brain regions’ activity over time and is usually interpreted as the propensity with which two brain regions communicate with one another^4^. In line with this interpretation, each layer in the multi-layer representation can be viewed as an estimate of the instantaneous communication pattern among brain regions^8^. Next, we used a community detection algorithm to partition brain regions into communities across layers (windows)^20^ (see Fig. 1 for a schematic illustrating the processes of network construction, multi-layer network model, community detection, and flexibility estimation). Intuitively, communities can be thought of as segregated sub-networks: brain regions assigned to the same community are more likely to be strongly connected to one another compared to regions assigned to different communities^19^. In the context of human brain networks, communities represent the boundaries between cognitive sub-systems^14^ and are believed to promote functional specialization^17^. Based on these detected community assignments, we calculated the flexibility of each brain region as the fraction of times that its community assignment changed from one layer to the next^12^. Flexible brain regions were those that frequently changed their community assignment from one layer to the next, whereas inflexible brain regions maintained consistent community assignments across layers. We repeated this analysis for all scan sessions.

### Somatomotor, visual, and fronto-parietal systems are inflexible but variable from day to day

**Figure 2:**
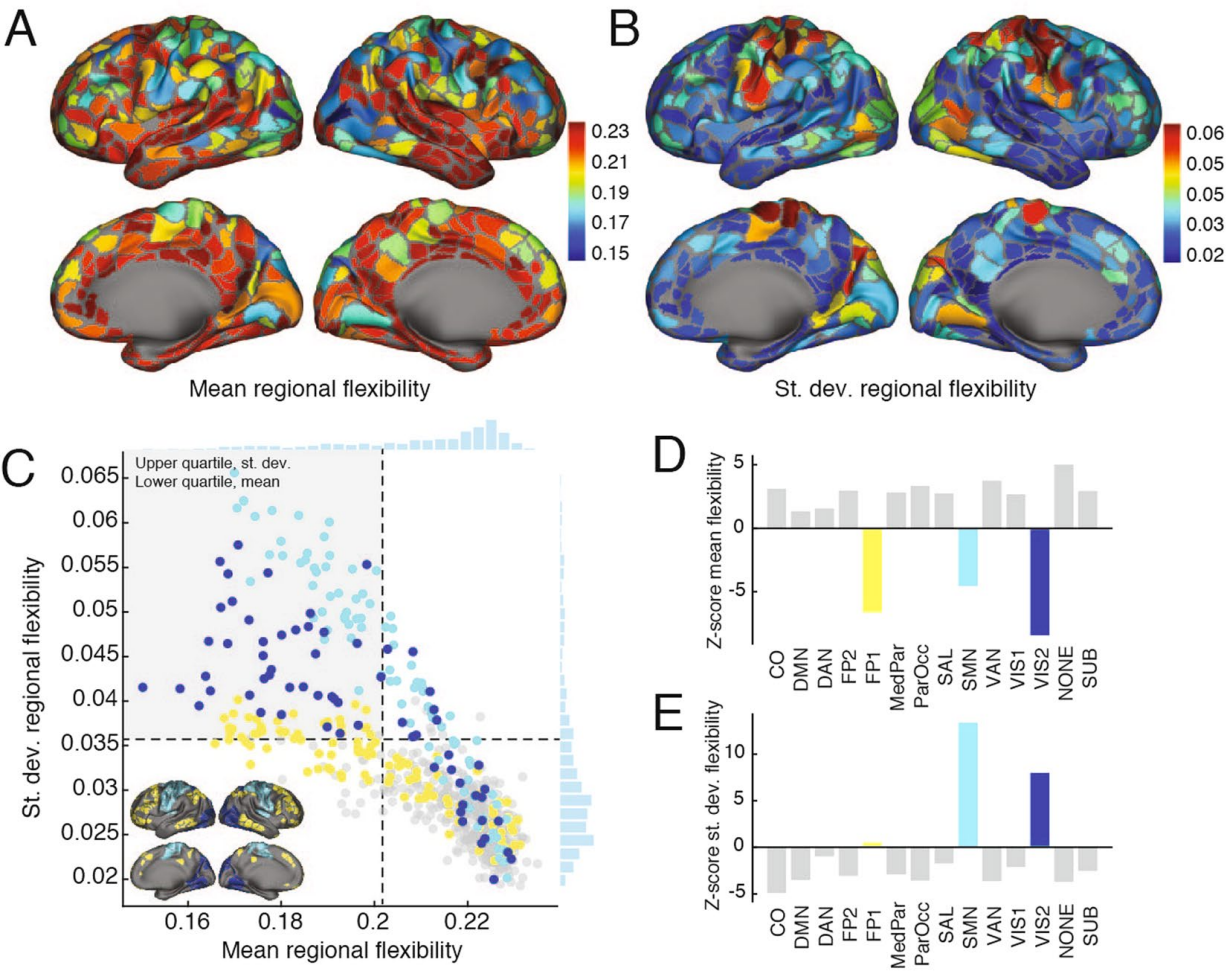
Summary of flexibility analysis. (**A**,**B**) Topographic representation of mean and standard deviation flexibility. (**C**) Mean and standard deviation of flexibility scores plotted against one another. Color-coding indicates brain systems: fronto-parietal (purple), somatomotor (green), peripheral visual (blue), and the rest of brain (grey). Inset depicts topographic representation of somatomotor (SMN), fronto-parietal (FP1), and peripheral visual (VIS2). (**D**) The *z*-score of the mean system flexibility. (**E**) The *z*-score of the standard deviation of system flexibility.

We first sought to determine which regions of the brain were flexible *versus* inflexible, and which regional flexibility values varied appreciably across scan sessions. We observed that mean and standard deviation of flexibility across scan session fell within a narrow range for most brain regions (Fig. 2A,B). A small number of regions, however, including components of visual, fronto-parietal, and somatomotor systems, possessed lower mean flexibility and greater variability of flexibility than the rest of the brain (Fig. 2C). To quantify these observations and to make concrete statements about specific brain systems, we aggregated regional flexibility by cognitive system and found that these same systems had mean flexibilities much lower than expected by chance (permutation test, *z*_*FP*2_ = −6.52, *p* = 3.44 × 10^−11^; *Z*_*SMN*_ = −4.53, *p* = 3.00 × 10^−6^; *z*_*VIS*2_ = −8.52, *p* < 10^−15^; FDR-controlled, *d* = 0.001) (Fig. 2D). Similarly, the quotidian variability of SMN and VIS2 were much greater than expected by chance (permutation test, *z*_*SMN*_ = 13.45, *p* < 10^−15^; *z*_*VIS*2_ = 7.93, *p* < 10^−15^; false discovery rate controlled at *d* = 0.001) (Fig. 2E). These observations corroborated earlier analyses of these data in which somatomotor and visual systems exhibited the greatest variability in their connectivity patterns across scan sessions estimated outside of mesoscale network statistics^34^. The presence of high quotidian variability in relatively rigid regions suggests the presence of a strong energetic constraint on network dynamics. Rigid regions are strongly functionally connected to one another, requiring high metabolic resources^36^; if these resources are not required by the cognitive tasks of a particular day, strong coupling might be used sparingly and transiently, possibly leading to high levels of network flexibility.

### Dimension reduction and correlation of PANAS-X scores with global flexibility

**Figure 3:**
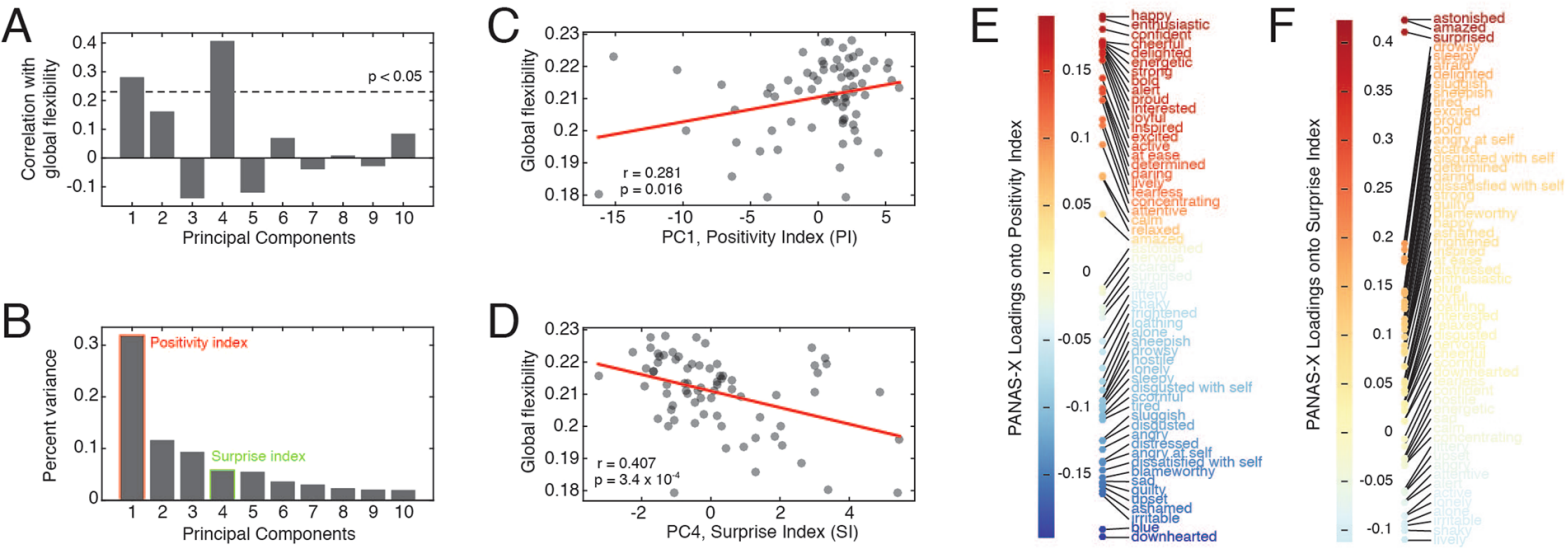
Relationship of global flexibility to principal components. (**A**) Correlation of first ten principle components with global flexibility. (**B**) Percent variance accounted for by each of said components. (**C**) Scatterplot highlighting relationship of the first principal component (PC1) with global flexibility. (**D**) Scatterplot highlighting relationship of the fourth principal component (PC4) with global flexibility. (**E**) Detail of the positivity index (PC1). Each colored point indicates the loading of a PANAS-X category onto the first principal component, which we termed the “positivity index”. (**F**) Detail of the surprise index (PC1). Each colored point indicates the loading of a PANAS-X category onto the fourth principal component, which we termed the “surprise index”. Note: In panel (A) the correlation of PC4 and flexibility was positive. For ease of interpretation, we flipped the sign of PC4 and the loadings of PANAS-X terms onto that component. In effect, this only changes the sign of the correlation; not its magnitude.

Concurrent with the collection of neuroimaging data, the participant also underwent a battery of behavioral testing. These tests included the administration of a standard mood questionnaire (the expanded Positive and Negative Affect Schedule; PANAS-X)^37^, which tallied subjective ratings across 60 mood terms using a 0–5 Likert scale (See Supplementary Table S1 for a complete list of all terms). We calculated the correlation of ratings across data collection sessions and observed that many of the terms were highly correlated with one another, suggesting that the data could be represented with fewer dimensions. We interrogated this structure using principal components analysis, which is a dimensionality reduction technique that generated a set of mutually orthogonal principal components, loadings of PANAS-X terms onto components, and the percent variance accounted for by each component. We tested the hypothesis that flexibility is associated with mood by calculating the Pearson’s correlation of global flexibility, the average flexibility across all brain regions, with each of the principal components, the first (PC1) and fourth (PC4) of which exhibited statistically significant correlations (Pearson correlation: $\hat{r}(P{C}_{1},F)=0.289$, $p=0.013$; $\hat{r}(P{C}_{4},F)=-0.407$, $p=3.5\times {10}^{-4}$; Spearman correlation: $\hat{\rho }(P{C}_{1},F)=0.203$, $p=0.084$; $\hat{\rho }(P{C}_{4},F)=-0.400$, $p=0.0006$) (Fig. 3A,C,D). PC1 accounted for ≈33 percent of the variance (Fig. 3B). The top five terms, in terms of the loading magnitude onto PC1, were “happy”, “enthusiastic”, “confident”, “cheerful”, and “delighted”. The bottom five were “downhearted”, “blue”, “irritable”, “ashamed”, and “upset”. Because of its apparent sensitivity to emotional valence, we termed the first principal component the “positivity index” (*PI*) (Fig. 3C). PC4 accounted for ≈6 percent of the variance. The top loadings were “suprised”, “amazed”, and “astonished”, leading us to term this component the “surprise index” (*SI*) (Fig. 3D).

### Correlation of global flexibility with positivity and surprise indices is driven by somatomotor system

**Figure 4:**
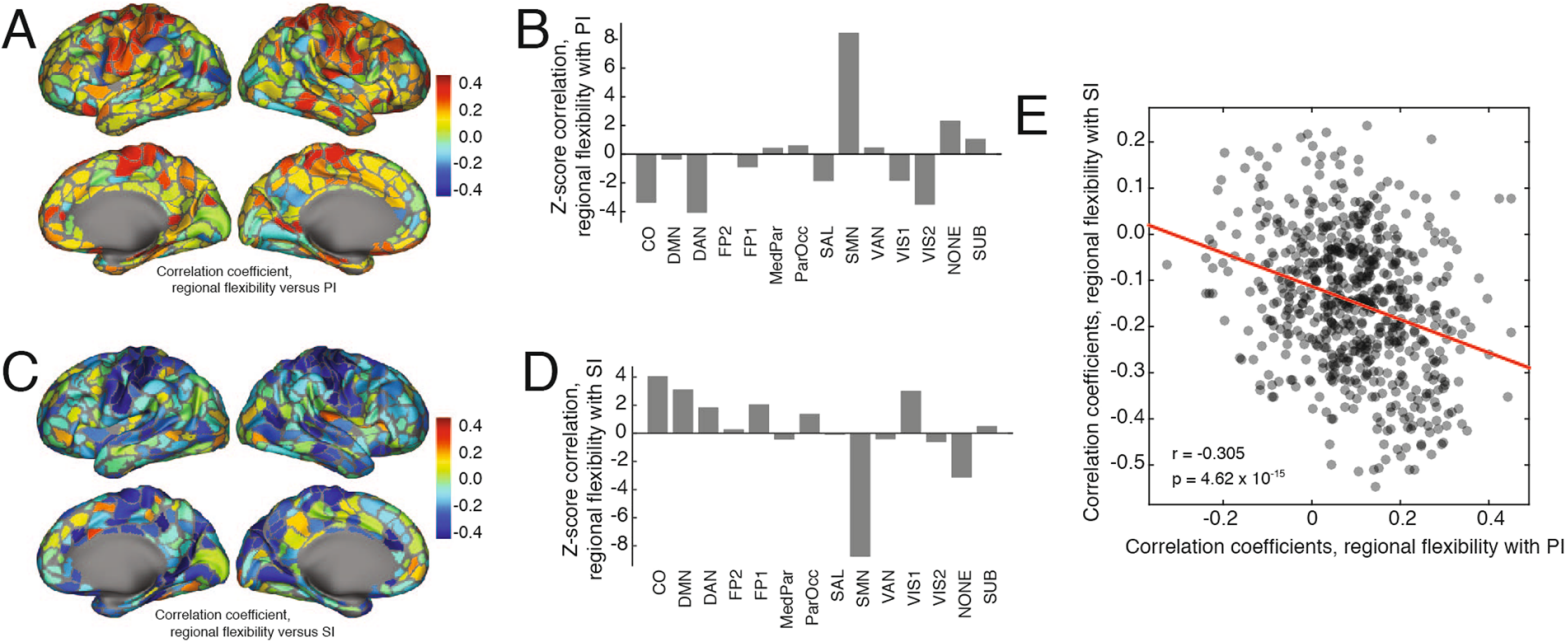
Relationship of regional flexibility with positivity and surprise indices. (**A**) Topographic representation of the correlation coefficient of each region’s flexibility score with the positivity index. (**B**) Regional correlations grouped by system. Panels (**C,D**) are identical to (**A,B**), but replace the positivity index with the surprise index. (**E**) Regional correlation coefficients – of flexibility with positivity and flexibility with surprise – plotted against one another.

We observed that global flexibility was correlated with both positivity and surprise indices. Which brain regions drive these correlations? To address this question we calculated the correlation of regional flexibility scores, measurements of how frequently each brain region changes its community assignment across windows, with both positivity and surprise indices. We observed that the flexibility of regions comprising the somatomotor system exhibited significant positive correlations with the positivity index (permutation test, *z*_*PI*_ = 8.49, *p* < 10^−15^) (Fig. 4A,B). Similarly, we observed that the flexibility of somatomotor regions were also anti-correlated with the surprise index (permutation test, *z*_*SI*_ = −8.76, *p* < 10^−15^) (Fig. 4C,D). The spatial specificity of these relationships complement prior work linking heightened motor activations with positive mood^38^ and work demonstrating a slowing down of action/motor processing in response to unexpected and surprising events^39^.

We also observed that two cognitive systems exhibited significant anti-correlations with the positivity and surprise indices. The dorsal attention system (DAN) was anti-correlated with the positivity index (*z*_*DAN*_ = −4.16, *p* = 1.56 × 10^−5^, FDR-controlled, *d* = 0.001), and the cingulo-opercular (CO) system was correlated with the surprise index (*z*_*CO*_ = 4.08, *p* = 2.26 × 10^−5^, FDR-controlled, *d* = 0.001). These results suggest that relative stability in higher-order cognitive systems affiliated with online control is accompanied by positive mood and decreased levels of surprise, offering a dynamic complement to the known relationship between control performance and affect^40^.

These results were corroborated by a series of additional analyses (See Supplementary Information). First, the correlations of *PI* and *SI* with flexibility were robust to variation in the parameters of our community detection method (Fig. S2). Second, these results were not driven by anomalous data points and data collection sessions (Fig. S3) and were not likely to have been observed given a random ordering of data collection sessions (Fig. S4). Third, we also observed consistent correlations when we focused on the relationship of flexibility to individual PANAS-X terms and pre-defined “affect classes” (Figs S5–S7)^41^. Finally, we tested the robustness of our results after controlling for in-scanner head motion (Fig. S8) as well as psychophysiological and other nuisance variables (Fig. S9). We also observed similar findings using window sizes of different durations (Fig. S11). In general, these supplementary analyses buttress the finding of a significant positive association between network flexibility and *PI*, and a negative association between network flexibility and *SI*.

### Discounting fatigue as a potential driver of flexibility

**Figure 5:**
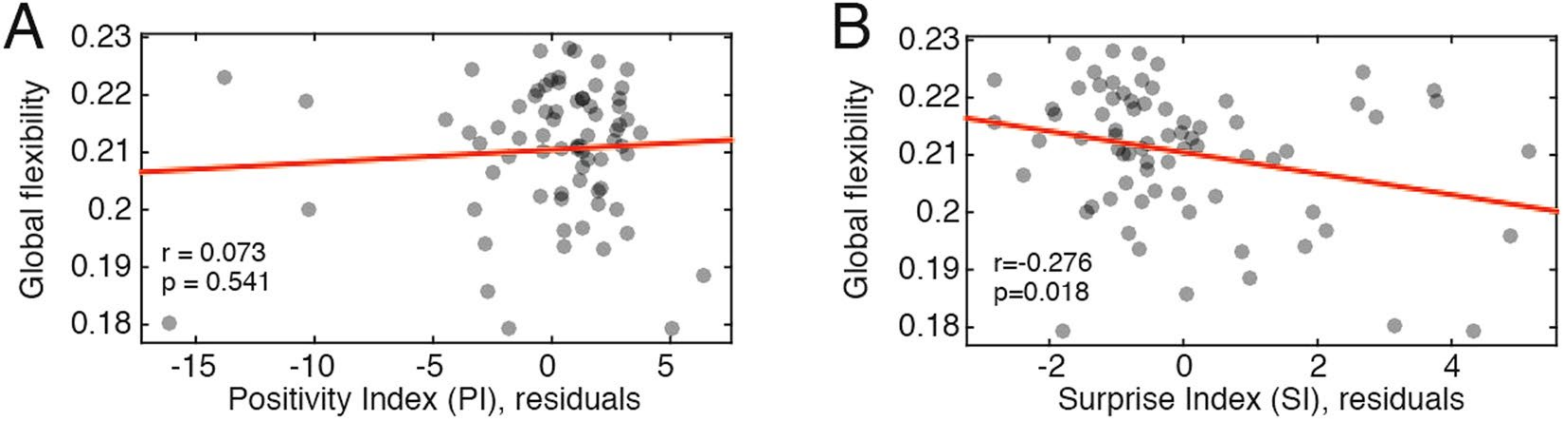
Correlation of positivity and surprise indices after controlling for fatigue. (**A**) Scatterplot of global flexibility with residuals of the positivity index after regressing out fatigue. (**B**) Scatterplot of global flexibility with residuals of the surprise index after regressing out fatigue.

Initial analyses of *MyConnectome Project* data described an effect wherein visual and somatomotor variability could be explained on the basis of fatigue^34^, a finding that has been reported elsewhere^42^. Accordingly, we assessed whether the correlations of positivity and surprise with flexibility could be driven by fatigue. As a measure of fatigue, we combined the PANAS-X categories “sleepy”, “tired”, “sluggish”, and “drowsy” into a “fatigue” affect score^41^. This score was correlated with both the positivity and surprise indices ($\hat{r}(fatigue,PI)=-0.48$, *p* = 1.82 × 10^−5^; $\hat{r}(fatigue,SI)=-0.32$, *p* = 0.0057). To test whether the observed correlation of *PI* and *SI* with global flexibility could be attributed to *fatigue*, we regressed *fatigue* from both indices and calculated the correlation of global flexibility with the residuals (Fig. 5). We observed that after regressing out *fatigue* from the positivity index, the residuals were no longer correlated with global flexibility at a statistically significant level ($\hat{r}(F,PI\backslash fatigue)=0.07$, *p* = 0.541), indicating that the correlation of *PI* and flexibility could be attributed to *fatigue*, in agreement with extant literature. On the other hand, after regressing *fatigue* from the surprise index, we observed that the residuals were still correlated with flexibility ($\hat{r}(F,SI\backslash fatigue)=0.276$, *p* = 0.018). Collectively, these results indicate that fatigue and affect (both level of positivity and surprise) represent powerful potential drivers of network flexibility. They also suggest that positivity may be confounded with the subject’s fatigue level, making it difficult to disambiguate whether positivity makes an independent contribution to network flexibility. Surprise, on the other hand, was still significantly associated with flexibility, even after controlling for fatigue, suggesting that it may independently drive flexibility.

## Discussion

The relationship between affect and flexibility suggests a potential network-level mechanism for learning deficits observed in mood disorders^43^, and the dependence of those deficits on cognitive flexibility^44^. In these individuals, the development of pharmacological and stimulation-based interventions to alter brain network flexibility is therefore of particular interest. For example, brain network flexibility can be altered through modulation of NMDA receptor function^45^. However, an arguably more powerful approach might be to target states of arousal, which are known to be altered in mood disorders^46^, implicated in learning^25^, and modulated by norepinephrine systems^25^. There is some preliminary evidence that arousal modulates network connectivity^26^, but further work is needed to understand the patterns and dynamics of these network modulations and their relationship to mood.

We observed that the subject’s level of surprise was negatively correlated with flexibility, indicating that decreased surprise is accompanied by increased network variability. This observation is similar to how complex motor behaviors are learned; during early learning when the movement is novel and unfamiliar, there is sometimes a “freezing” period during which only a small number of the system’s degrees of freedom are actively manipulated; with increased familiarity, those components are gradually “freed” and can be incorporated into the movement pattern^47, 48^. Here, we speculate that the relationship of surprise to flexibility is analogous to the relationship of novelty to motor variability. Indeed, surprise and novelty are sometimes viewed as related concepts^49^, and furthermore, our findings directly implicate the somatomotor network as the system that most closely tracks surprise. To investigate the complicated relationship of flexibility, learning, and surprise, future work could involve the study of motor learning paradigms similar to those described elsewhere^12^ but supplemented with administration of PANAS-X questionnaires to track surprise and other affective components, particularly contrasting effects with and without positive(negative) mood induction.

An alternative hypothesis is that the negative correlation of surprise and flexibility reflects the “inverted-U” relationship of learning with arousal and surprise. Here, it may be the case that self-reports of surprise fall on the right side of the “U” and correspond to the high end of arousal, which can lead to increased levels of distraction that hamper learning^24^. Future work is needed to compare these two hypotheses.

Our observations can also inform the development of educational interventions to enhance learning. Intuitively, our results support the notion that by altering mood, one might alter brain network flexibility, and therefore predispose the brain to learn quickly in subsequent tasks. Such an outcome would directly fulfill the goals of personalized neuroeducation^50^: the use of neuroscientific information to inform educational practices tuned to individual students. Potentially powerful modulations could include simple mental exercises, which are easily translated into educational settings. For example, self-affirmation tasks have been shown to parametrically alter brain activity^51^ to a degree that predicts individual differences in future behavior^52^. Future work could define a carefully titrated library of mental tasks that modulate brain network flexibility (and subsequent learning) in a predictable fashion by modulating mood.

While our study informs our understanding of how the human brain is related to human behavior, it is also important to bear in mind that extrapolating our findings beyond the present study is hampered by the fact that we study data recorded from one subject. Indeed, recent studies have shown that the modular and system-level neural architecture of the brain varies across individuals in idiosyncratic ways^53–55^, suggesting the possibility that network flexibility could do the same. Future studies should investigate whether the findings reported here are general and broadly applicable to large populations of individuals, or whether they apply more narrowly to the individual studied here.

Finally, our results inform the field of network neuroscience^56^, more broadly, where the typical analysis has sought network features that can be used to classify brains at the population level – e.g. healthy *versus* pathological^57–59^, young *versus* old^60, 61^, or task *versus* task-free^62^. While such classifications have provided insight into the average network organization of the human brain, they nonetheless overlook potentially meaningful individual variation^63^. Indeed, recent work has demonstrated that functional brain networks encode nuanced, personalized features of an individual^64, 65^ that moreover can change with an individual’s cognitive state^66^. Our results build on and contribute to this growing body of literature, identifying network flexibility in the somatomotor cortex as a potential neuromarker of mood. With personalized medicine and healthcare increasingly becoming a reality, such subject-level markers may serve as important measures to facilitate diagnosis, target interventions, and monitor disease progression or response to treatment^67^.

## Materials and Methods

### MyConnectome data

All data and cortical surface files are freely available and were obtained from the *MyConnectome Project*’s data-sharing webpage (http://myconnectome.org/wp/data-sharing/). Specifically, we studied pre-processed parcel fMRI time series for scan sessions 14–104. Details of the pre-processing procedure have been described elsewhere^34, 35^. Each session consisted of 518 time points during which the average fMRI BOLD signal was measured for *N* = 630 parcels or regions of interest (ROIs). With a TR of 1.16 s, the analyzed segment of each session was approximately 10 minutes long. In addition to fMRI data, we also examined behavioral data available on the same webpage. The behavioral data included additional biometric information, such as blood pressure and sleep quality as well as variations in temperature and weather (See Supplementary Table 2 for a complete list). We analyzed only PANAS-X terms, a set of emotional terms that the subject rated on a 0–5 Likert scale. Usually the PANAS-X test includes 60 terms. Only 57 were used as part of our analysis; the terms “bashful”, “timid”, and “shy” had ratings of zero for the entire duration of the experiment.

### Principal component analysis

Our analysis focused on the *n* = 73 scan sessions for which both resting-state fMRI data and all *p* = 57 PANAS-X terms were available. We standardized each category to have zero mean and unit variance. We represented the full set as the matrix, ${\bf{X}}\in {{\mathbb{R}}}^{n\times p}$, which we submitted to a principal component analysis (PCA). Essentially, PCA takes a data matrix and linearly factorizes it by creating a set of orthogonal *principal components*, subject to the condition that each successive component has the greatest possible variance. Each component is a linear combination of the original data variables.

Specifically, we performed PCA using a singular value decomposition (SVD)^68^ which deconstructs **X** according to the equation:

1 $${\bf{X}}={\bf{US}}{{\bf{V}}}^{T}$$

where ${\bf{U}}\in {{\mathbb{R}}}^{n\times n}$ and ${\bf{V}}\in {{\mathbb{R}}}^{p\times n}$ contain the left and right singular vectors and where ${\bf{S}}\in {{\mathbb{R}}}^{p\times p}$ is the diagonal matrix of singular values. Importantly, **U** and **V** have rank equal to that of **X**. The *i*th principal component, then, is the *i*th column of **U**. The corresponding column of **V** gives weights that indicate the extent to which each PANAS-X category contributed to that component. Similarly, squaring the corresponding singular element of **S** gives the magnitude of variance accounted for by that component.

### Dynamic network construction and community detection

We sought a division of brain regions into communities, which are thought to reflect the brain’s functional sub-systems^19^. We divided the parcel time series into *T* = 14 windows of 37 time points (TRs) each (≈43 seconds in length). This particular window length was chosen for several reasons. First, recent work has emphasized that short window lengths (much less than 40 s) may result in time-varying FC matrices in which the connectivity pattern is driven predominantly by sampling variability rather than true fluctuations in network organization^69, 70^. Accordingly, one of our aims was to ensure that the length of our windows met this minimum requirement. Second, rather than estimate FC using linear correlations of BOLD activity, we used wavelet coherence, a measure that has at least as much behavioral and neurobiological relevance as linear correlation^71^ but benefits from longer samples^72^. Finally, we wanted to ensure that whatever window length we selected would evenly divide into the 518 samples (TRs) collected for each scan session. Collectively, these three factors – methodological and practical – motivated our selection of the 37 TR window size.

For each window, we calculated the wavelet coherence matrix, ${\bf{C}}\in {{\mathbb{R}}}^{N\times N}$ ^73^. Each element, *C*_*ij*_, represented the magnitude squared coherence of the scale two Daubechies wavelet (length 4) decomposition of the windowed time series obtained from regions *i* and *j* (http://www.atmos.washington.edu/wmtsa/)^12^. We examined two frequency ranges: 0.125–0.25 Hz, the results of which we describe in the main text, and 0.0625–0.125 Hz^12, 23^, which are covered in the supplementary materials (Fig. S10).

Each dynamic network was treated as a layer in a multi-layer network, ${\mathscr{C}}=\{{{\bf{C}}}_{1}\ldots {{\bf{C}}}_{14}\}$ ^74^. To detect the temporal evolution of modules, we maximized the multi-layer modularity^20^, which seeks the assignment of all brain regions in all layers to modules such that:

2 $$Q(\gamma ,\omega )=\frac{1}{2\mu }\sum _{ijsr}[({C}_{ijs}-\gamma {P}_{ijs})]\delta ({G}_{is},{G}_{js})+\delta (i,j)\cdot \omega ]\delta ({G}_{is},{G}_{jr})$$

is maximized. In this expression, *C*_*ijs*_ is the coherence of regions *i* and *j* in layer *s*. The tensor *P*_*ijs*_ is the expected coherence in an appropriate null model. Specifically, we choose ${P}_{ijs}=\frac{{k}_{is}{k}_{js}}{2{m}_{s}}$, which is a multi-layer extension of the common configuration model (see Refs. 75–77 for further discussions on the choice of null model). The parameter, *γ*, scales the relative contribution of the expected connectivity and effectively controls the number of modules detected within a given layer. The other free parameter, *ω*, defines the weight of the inter-layer edges that link each node *i* to itself across layers. Effectively, the value of *ω* determines the consistency of multi-layer modules; when its value is large (small) relative to the intralayer links, the detected modules will tend to be more (less) similar to one another across layers. For this reason, *ω* is sometimes referred to as the *temporal resolution parameter* ^21^. In the main text, we fix these parameters to the commonly used default values of *γ* = *ω* = 1^21^.

We use a Louvain-like locally greedy algorithm to maximize the multi-layer modularity, *Q*(*γ*, *ω*)^78^ (typical output is show in Fig. S1). Due to near-degeneracies in the modularity landscape^79^ and stochastic elements in the optimization algorithm^80^, the output typically varies from one run to another. For this reason, rather than focus on any single run or the consensus communities over many runs^81^, we characterized the statistical properties of 50 runs of the algorithm, which correspond to 50 optimizations of the multi-layer modularity. Specifically, we calculated all network statistics, including network flexibility, for each run of the community detection algorithm and subsequently averaged those statistics over the 50 runs to obtain an estimate of their mean value.

#### Regional and global flexibility

The output of the Louvain-like locally greedy algorithm is a partition, ${\bf{G}}\in {{\mathbb{R}}}^{N\times T}$, whose element ${G}_{i,r}\in \{1\ldots c\}$ is the community to which brain region *i* in layer *r* is assigned in that optimization. The multi-layer modularity maximization simultaneously assigns brain regions in all layers to communities so that community labels are consistent across layers, thus circumventing the commonly studied community matching problem. Given **G**, we can calculate each brain region’s flexibility score:

3 $${f}_{i}=1-\frac{1}{T-1}\sum _{s=1}^{T-1}\delta ({G}_{i,s},{G}_{i,s+1}),$$

which counts the fraction of times that brain region *i* changes its community assignment in successive layers. Flexibility is normalized so that scores near zero and one correspond to brain regions whose community assignments are highly consistent and highly variable, respectively, across layers. Flexibility can also be averaged over all brain regions to obtain the *global flexibility* of the whole brain, $F=\frac{1}{N}{\sum }_{i=1}^{N}{f}_{i}$. Both regional and global flexibility scores were calculated separately for each of the 50 modular partitions obtained from the Louvain-like algorithm and averaged across optimizations.

Intuitively, the flexibility measure serves as an indicator of community stability across layers, which represent time, in this case. Regional flexibility scores are bounded between 0 and 1; a value near 0 implies that a node’s community assignment varies little over the course of a scan session while a value close to 1 implies that a node’s community assignment is highly variable. The global flexibility score can be interpreted similarly.

## Electronic supplementary material

Supplementary information
