# Supplementary material for: Positive affect, surprise, and fatigue are correlates of network flexibility
Source: Sci Rep. 2017 Mar 31;7:520. doi: 10.1038/s41598-017-00425-z (PMC5428446; doi:10.1038/s41598-017-00425-z)
Supplement: Supplementary file 1 — Supplementary information [file 41598_2017_425_MOESM1_ESM.pdf]

Supplementary Materials for "Positive affect, surprise, and fatigue are correlates of network flexibility"

Richard F. Betzel,[1] Theodore D. Satterthwaite,[2] Joshua I. Gold,[3], and Danielle S. Bassett[1,4*]

[1]Department of Bioengineering, University of Pennsylvania,

[2]Neuropsychiatry Section, Department of Psychiatry, University of Pennsylvania

[3]Department of Neuroscience, University of Pennsylvania

[4]Department of Electrical and Systems Engineering, University of Pennsylvania

Philadelphia, PA, 19104, USA

[*]To whom correspondence should be addressed; E-mail: dsb@seas.upenn.edu.

Contents of this Supplementary Document

This file includes the following content:

1. Supplementary Materials and Methods

2. Figures S1 to S10

3. Tables S1 to S3

4. References

1 Supplementary Materials and Methods

1.1 Cognitive systems

In the main text we used a multi-layer community detection algorithm to uncover brain network modules across layers (windows). In addition to these detected modules, each brain region was also assigned to a brain system based on a previous study (*1*). That study identified, in total, 13 systems: cingulo-opercular (CO), default mode (DMN), dorsal attention (DAN), fronto-parietal 1 (FP1), fronto-parietal 2 (FP2), medial-parietal (MedPar), parietal-occipital (ParOcc), salience (SAL), somatomotor (SMN), ventral attention (VAN), primary visual (VIS1), and peripheral visual (VIS2). In addition to the previously-defined systems (*1*) we also included two other systems: (i) a subcortical (SUB) system comprised of bilateral thalamus, caudate, putamen, pallidum, hippocampus, amygdala, and accumbens, and (ii) a category reserved for brain regions with no clear assignment (NONE) (See Fig. S1).

In the main text we sometimes found it advantageous to describe certain measures (e.g. mean and standard deviation of flexibility; correlation of regional flexibility with positivity and surprise indices) at the intermediate level of cognitive systems rather than at the level of individual brain regions or at the level of the whole brain. Such system-level measurements were obtained by averaging the measure-in-question across each system's constituent regions. However, because such measures may be biased by system size (i.e. number of regions assigned to that system), we compared the observed measures against the distribution of similar measurements obtained from a permutation null model, wherein the total number of regions assigned to each system remained constant but where assignments were, otherwise, made at random. Specifically, we calculated the mean, μ_{sys}, and standard deviation, σ_{sys}, system-level measurements based on 10000 iterations of the permutation null model and expressed the observed measure, y_{sys}, as a z-score:

$$z_{sys} = \frac{y_{sys} - \mu_{sys}}{\sigma_{sys}}. \quad (1)$$

We corrected for multiple comparisons by controlling the false discovery rate (FDR) using the linear step-up procedure (*2*). In each case, we calculated an adjusted critical value, p_{adj}, by fixing the maximum FDR at $d = 0.001$.

It should be noted that we rely on a particular definition of cognitive systems based on a previously published study (*1*). While this same set of systems has been exploited in other studies (*3, 4*), it is worth noting that there are alternative methods for defining such systems, which could produce somewhat different results. For example, in the present study, each subcortical region is represented by a single network node and all subcortical regions are grouped together to form a cohesive cognitive system. While this definition is well-suited for the exploratory nature of the study, future work could investigate, in more detail, the nuanced connectivity patterns of sub-cortical structures (e.g., hippocampus), and further sub-divide these regions based on those patterns. Investigations of this type, however, require specialized analysis tools and therefore we leave this for future work.

1.2 Robustness to choice in resolution parameters

The multi-layer modularity maximization equation included two free parameters: γ and ω. Together with the structure of the multi-layer network, itself, these parameter determine the composition of the communities detected by maximizing modularity (*5*). Though inexact, we can think of γ as controlling the number and size of communities and ω as controlling the consistency of communities across layers. Depsite the importance of these parameters, they are often fixed at the *de facto* values of $\gamma = \omega = 1$ (*6*). Though a complete exploration of both parameters is beyond the scope of most studies, it is considered good practice to demonstrate that one's results are robust to reasonable variations in the parameters' values (*6–9*).

Accordingly, we sought to replicate the principal results from the main text, i.e. the correlation of flexibility with positivity and surprise indices, across variations in the two resolution parameters. To this end, we varied both γ and ω over the range [0.95, 0.975, 1.00, 1.025, 1.05] and maximized multi-layer modularity for all pairs of parameter values. This procedure generated 25 estimates of $\hat{r}(PI, F)$ (24 *new* estimates, including a repetition of the case where $\gamma = \omega = 1$, which was already investigated in the main text). In general, these results supported the hypothesis that $\hat{r}(PI, F) > 0$ (Fig. S2A) and $\hat{r}(SI, F) < 0$ (Fig. S2B.

Exploring the parameter landscape facilitated an investigation into the properties of the detected communities that were responsible for driving the relationships of positivity and surprise with flexibility. We focused, in particular, on the total number of communities present across all layers and the average number of communities present in any given layer. We calculated the mean values of these variables across all detected communities and compared these values to the behavior-flexibility correlation magnitudes at different values of γ and ω (Fig. S2C-F). We found that correlation magnitude varied systematically with both variables, with $\hat{r}(PI, F)$ exhibiting a peak between 3–4 communities and $\hat{r}(SI, F)$ peaking around 4–5 communities. This finding suggests that the observed behavior-flexibility correlations depend upon the scale at which we interrogate the network's community structure. Moreover, it further reinforces the hypothesis that the brain is a multi-scale organ that demands appropriate multi-scale tools for characterizing its organization and relationship to cognition and behavior (*10*).

1.3 "Leave one out" analyses

Pearson's correlation coefficient, which we used to assess the relationship of flexibility with positivity and surprise indices, can be driven by outlying data points. Therefore we wanted to test whether the reported correlations could be driven by either a single data collection session or PANAS-X category. To test whether this was the case, we performed a series of "leave one

out" analyses, wherein we iteratively removed either PANAS-X categories or data collection sessions, performed PCA on the limited dataset, and then calculated the correlation of flexibility with the new estimates of the positivity and surprise indices. These supplemental analyses supported the hypotheses that $\hat{r}(PI, F) > 0$ (Fig. S3A,B) and $\hat{r}(SI, F) < 0$ (Fig. S3C,D). These results indicate that our results are unlikely to be biased by single data collection sessions or PANAS-X categories.

1.4 Permutation tests for confidence intervals

In the main text we presented evidence suggesting that specific brain regions drive the correlation of PI and SI with flexibility, namely regions associated with somatomotor cortex. We arrived at this conclusion based on the observation that the regional flexibility of somatomotor regions were highly correlated with both PI and SI. Given two sets of observations, even random orderings can sometimes produce large correlation coefficients. Therefore, we wished to test the hypothesis that the observed correlation magnitude of PI and SI with flexibility could arise simply by chance. To do so, we randomly permuted the order of both global and regional flexibility scores, and recalculated the Pearson's correlation of the reordered flexibilities with PI and SI. We repeated this procedure 10000 times in order to obtain null distributions for the correlation coefficients $\hat{r}(PI, F)$ and $\hat{r}(SI, F)$ and also for the correlation coefficients of each regional flexibility with PI and SI ($p = 0.014$ and $p = 0.0001$ for PI and SI, respectively) (Fig. S4A,B).

We performed a similar test at the level of brain regions, standardizing observed correlation coefficients against the null distribution and representing them as z-scores. In general, patterns of z-score coefficients closely resembled those shown in the main text (Fig. S4C,D). Moreover, when aggregated by cognitive systems, the z-score coefficients were greatest for the somatomotor system, also in agreement with the results presented in the main text (Fig. S4E,F).

Collectively, the results of this section demonstrate that the observed correlation of PI and SI with flexibility are not likely to have been driven by random orderings of the flexibility scores and that, once again, these correlations are strongest in the somatomotor system.

1.5 Correlation of individual PANAS-X scores with flexibility

The results presented in the main text showed that global flexibility was positively correlated with the positivity index and negatively correlated with the surprise index. Both indices were derived from a PCA of PANAS-X terms. While our analyses in the main text focused on these components, we thought that it would be illuminating to present the reader with plots showing the relationship of individual PANAS-X terms with global flexibility. In Fig. S5 we show the PANAS-X terms according to the magnitude of their loadings onto the positivity and suprise indices. In the top row of Fig. S5 (panels *A*-*C*), we show the top three terms: *happy*, *enthusiastic*, and *confident*. The bottom three terms are shown in the middle row (Fig. S5D-E): *irritable*, *downhearted*, and *blue*. As expected, the terms in the top row with strong positive loadings on the positivity index also tended to be positively correlated with flexibility. Similarly, the terms in the middle row with negative loadings tended to be negatively correlated with flexibility. In the bottom row (Fig. S5F-H) we show the top three components that load onto the suprise index: *amazed*, *surprised*, and *astonished*. Predictably, there terms tended to be positively correlated with global flexibility. In the case of the surprise index, no terms exhibited strong negative loading.

1.6 PANAS-X affect scores

The PANAS-X includes $p = 60$ terms (we studied a subset of 57 of these terms). In the main text we used a PCA to distill these terms into positivity and surprise indices. There are, however, other means of combing PANAS-X terms into composite indices. One stan-

dard grouping involves combining terms into the following twelve *affect classes*: "negative affect", "positive affect", "fear", "hostility", "guilt", "sadness", "joviality", "self-assurance", "attentiveness", "fatigue", "serenity", and "surprise" (*11*) (we show the class to which each term is assigned in *Supplementary Table S2*). Oftentimes an additional class, "shyness," is included; the terms comprising this class were omitted from this study. We tested whether global flexibility was correlated with affect classes. Indeed, a number exhibited statistically significant correlations with flexibility ($p < 0.05$; uncorrected). We observed that "positive affect" ($\hat{r} = 0.278, p = 0.017$), "guilt" ($\hat{r} = -0.268, p = 0.022$), "joviality" ($\hat{r} = 0.255, p = 0.029$), "attentiveness" ($\hat{r} = 0.324, p = 0.051$), "surprise" ($\hat{r} = -0.260, p = 0.026$), and "fatigue" ($\hat{r} = -0.455, p = 0.001$) were all correlated with flexibility (Fig. S6). In general, these results agree with those obtained from our PCA analysis, with "positive affect", "joviality", and "attentiveness" (which capture positive emotions, broadly) all exhibiting positive correlations with flexibility. Interestingly, the PANAS-X terms that comprise the "surprise" class were identical to those with the strongest loadings onto the fourth principal component, which we termed the surprise index, and exhibited a statistically significant positive correlation ($\hat{r} = -0.26$, $p = 0.026$) (Fig. S6L).

The affect class "fatigue" exhibited the greatest magnitude correlation with global flexibility. Accordingly, we investigated its correlation with regional flexibility scores to determine its topographic distribution and its mapping onto brain systems. Like the positivity and surprise indices, "fatigue" was strongly anti-correlated with the regional flexibility of somatomotor cortex ($z_{SMN} = -10.110$, $p_{SMN} < 10^{-15}$) (Fig. S7). Also as before, a number of other systems were also implicated, including cingulo-opercular (CO), fronto-parietal (FP1), and peripheral visual (VIS2) ($z_{CO} = 4.469$, $z_{FP1} = 4.481$, $z_{VIS2} = 3.002$; all $p < 0.001$), which were more positively correlated with fatigue than expected by chance.

1.7 Regressing out nuisance variables

To this point, we have demonstrated the robustness of the correlation between positivity (PI) and surprise (SI) indices with global flexibility, F. Another concern is that this relationship is mediated by a third, nuisance variable that we fail to account for. To determine whether this was the case, we investigated the relationship of F with head motion and other psychophysiological variables collected as part of the *MyConnectome Project*.

1.7.1 Relationship of global flexibility and head motion

Recent work has demonstrated that subject head motion within the scanner can introduce systematic biases in functional connectivity patterns (*12*). It is therefore possible that global flexibility, rather than tracking the reconfiguration of network communities over time, is driven by head motion. To test whether this was the case, we asked whether global flexibility was correlated with average frame-wise displacement, which represents an estimate of the amplitude with which a subject's head moves relative to a reference frame. Our analysis revealed no correlation between motion and global flexibility ($\hat{r}(\text{motion}, F) = 0.013$, $p = 0.912$).

We also performed two additional tests. First, we regressed motion from PI and SI and recalculated the correlation of the residuals with global flexibility (Fig. S8A,B) ($\hat{r}(PI \backslash motion, F) = 0.284$, $p = 0.015$; $\hat{r}(SI \backslash motion, F) = 0.407$, $p < 10^{-3}$). Second, we regressed motion from the global flexibility scores and calculated the correlation of those residuals with the original PI and SI (Fig. S8C,D) ($\hat{r}(PI, F \backslash motion) = 0.283$, $p = 0.015$; $\hat{r}(SI, F \backslash motion) = 0.406$, $p < 10^{-3}$). In both cases, accounting for motion had little effect on our reported results. This suggests that the relationships between flexibility and neuropsychological variables are unlikely to be driven by head motion.

1.7.2 Other psychophysiological measurements

In addition to PANAS-X terms, the *MyConnectome Project* made a number of other psychophysiological measures. For example, on certain recording sessions, blood was drawn and measures such as platelet and red blood cell counts made. Other measures included subjective rates of sleep quality, whether the subject drank alcohol the previous evening, and whether there was precipitation on the day of the scan (See TableS3 for a complete list). As with head motion, it was possible that fluctuations of these additional variables could explain the quotidian variability in flexibility.

As with the motion control section, we regressed each psychophysiological variable from F, and calculated the correlation of the residuals with PI and SI. Many of the psychophysiological measurements were not performed in every scan session. For such cases, we performed the regression analysis on the subset of sessions for which those variables were measured. In general, even after controlling for other psychophysiological variables, we still observed a positive correlation between PI and F (Fig. S9A) and a negative correlation between SI and F (Fig. S9B). In fact, regressing out most variables lead to little change in the overall correlations of both indices with flexibility. There are two notable cases, however, that deviate from this trend. First, after controlling for variables based on bloodwork (in Fig. S9C they are preceded with the label "blood:") we observed a large increase in the magnitude of $\hat{r}(PI, F)$ and a corresponding decrease in $\hat{r}(SI, F)$. However, bloodwork was performed on only 16 of the 73 analyzed recording sessions; such a small sample does not permit us to make strong quantitative statements. The second notable case concerned the variable "how much did tinnitus bother you today?". Controlling for this variable decreased $\hat{r}(PI, F)$ to ≈ 0.1. Like the bloodwork, this variable was measured during a small fraction of the recording sessions (22 of 73). Again, with such a small sample size we are not in a position to make strong quantitative statements about the relationship of the subject's tinnitus with flexibility. Limiting our-

selves to variables that were measured during at least 50% of the analyzed recording sessions, we found that the median correlation after regressing out each nuisance variable from F was comparable to the correlation magnitudes reported in the main text ($\hat{r}(PI, F)_{median} = 0.276$; $\hat{r}(PI, F)_{min} = 0.208$; $\hat{r}(PI, F)_{max} = 0.418$; $\hat{r}(SI, F)_{median} = 0.407$; $\hat{r}(SI, F)_{min} = -0.441$; $\hat{r}(SI, F)_{max} = -0.351$). While not conclusive, these results suggest that the psychophysiological measurements collected in addition to the PANAS-X scores did not, on their own, account for the strength of the correlation of flexibility with the positivity and surprise indices.

Another possible concern is related to the manner in which we measured the "fatigue" affect class, which we suggested might mediate the relationship between positivity and flexibility but not surprise and flexibility. The fatigue index that we analyzed was a composite score based on select PANAS-X categories (*11*). Accordingly, the fatigue index is not a direct measure of the subject's physiological state. Despite this, it has been shown to be reliable in that it tends to correlate with other psychological and physiological measurements of fatigue (*13*). Nonetheless, we felt that exploring alternative measures of fatigue was worthwhile.

In particular, we focused on the Zeo sleep data (Zeo, Inc., Boston, Massachusetts), which were collected using a single-channel EEG-like apparatus and generates data categories such as: "time in deep sleep", "time in light", "time in REM", "total sleep time", and "sleep quality". While the Zeo apparatus has been shown to do a reasonable job classifying sleep stage (*14, 15*), the veracity of these data in measuring physiological fatigue is unclear. Nonetheless, we can assess whether these data are related to the PANAS-X fatigue measure and also assess whether regressing sleep data from the positivity (or surprise) index weakens the correlation magnitude of either index with flexibility.

In general, we found that the sleep data were (i) not strongly correlated with the fatigue affect class, (ii) not strongly correlated with either the positivity or surprise indices, (iii) not strongly correlated with flexibility, and (iv) regressing the sleep data from those indices and

recomputing the correlation of the residuals with flexibility did not weaken the strength of the behavior-flexibility correlation. Specifically, the strongest correlation of sleep data and fatigue involved sleep quality $\hat{r}(\text{fatigue}, \text{“quality”}) = -0.12$, $p = 0.43$; the strongest correlation of sleep data and either the positivity or surprise indices was $\hat{r}(PI, \text{“time in light”}) = 0.11$,$p = 0.44$; the strongest correlation of sleep data with fatigue was $\hat{r}(\text{fatigue}, \text{“sleep quality”}) = -0.12$, $p = 0.43$; the weakest correlations after regressing sleep data from positivity or surprise were $\hat{r}(F, PI\backslash\text{“time in deep sleep”}) = 0.35$, $p = 0.018$ and $\hat{r}(F, SI\backslash\text{“total sleep time”}) = -0.40$, $p = 0.004$.

Collectively, these results indicate that the observed correlations of the positivity and surprise with flexibility are not likely mediated by the Zeo sleep scores. In general, we feel that these results should be viewed as complementary to those presented in the main text. While the fatigue index obtained from the PANAS-X data may not serve as a direct measure of physiological fatigue, it can represent fatigue induced by non-sleep factors. In contrast, the indices computed from the Zeo sleep data are collected using an EEG-like apparatus worn during sleep but do not account the subject's blood metabolic and chemical profiles. Furthermore, the Zeo sleep data was collected less frequently than the PANAS-X data, which weakens the strength of conclusions drawn from analyses using those data. In short, the PANAS-X data is a convenient, well-known, and reasonably reliable measure for assessing self-reported fatigue.

1.8 Analysis of low-frequency *versus* high-frequency fMRI BOLD signal

In the main text, we described correlative relationships between regional and global flexibility scores with two behavioral indices: a “positivity index” and a “surprise index.” These relationships were documented in fMRI BOLD signal sampled every 1.16 s and wavelet filtered into a high-frequency band (0.125–0.25 Hz). Analysis of high-frequency fMRI data is made possible with the faster-than-normal sampling rate. However, we also wished to stay consistent with past

work (*16, 17*), and so in parallel we pursued an identical analysis on data filtered using the more traditional frequency band of 0.0625–0.125 Hz. We performed the same procedures as those described in the main text:

1. Reconstructed dynamic functional connectivity matrices for each scan session.
2. Maximized a multi-layer modularity quality function to uncover community structure.
3. Calculated regional and global flexibility metrics from the community structure.

Broadly, these results corroborate those described in the main text. First, we calculated the similarity (Pearson correlation coefficient) of dynamic connectivity matrices in the low and high frequency bands. We observed a correlation of $\hat{r}(FC_{high}, FC_{low}) = 0.80 \pm 0.01$, suggesting that connections in one frequency band were similar to those in the other (Fig. S10A). Next, we performed multi-layer modularity maximization on the low-frequency connectivity matrices using precisely the same techniqes as described in the main text. Using the community estimates we then calculated global and regional flexibility. Again, we observed a close correspondence between the flexibility scores estimated in high and low frequency bands. Specifically, we observed a correlation of $\hat{r}(F_{high}, F_{low}) = 0.85$, $p < 10^{-23}$ for global flexibility and $\hat{r}(f_{high}, f_{low}) = 0.67 \pm 0.08$ for local flexibility across scan sessions, suggesting that flexibility scores were similar irrespective of which frequency band we studied (Fig. S10B,C). Finally, to demonstrate that we observe in the low frequency band the same behavior-flexibility relationships reported in the high frequency, we calculated the correlation of low frequency regional and global flexibility with the positivity and surprise indices. As in the main text, we observed statistically significant correlations: $\hat{\rho}(F, PC_1) = 0.20$ and $\hat{\rho}(F, PC_4) = -0.42$, both $p < 0.05$ (Fig. S10D-G). Moreover, and again in agreement the main text, if we regress *fatigue* from the positivity and surprise indices and recompute the correlations, we find that the correlation of flexibility with positivity is attenuated and no longer statistically significant while

the correlation of flexibility with suprise remains statistically significant using corrected data ($\hat{\rho}(F, PC_1 \backslash fatigue) = 0.02$, $p = 0.86$ and $\hat{\rho}(F, PC_4 \backslash fatigue) = 0.31$, $p = 0.007$). Finally, in order to demonstrate that the relationship between global flexibility and behavior was driven predominantly by the regional flexibility of the somatomotor network, we calculated the correlation of regional flexibility with both the positivity and surprise indices. In both cases, we found that the mean correlation of somatomotor regions' flexibility scores with the behavioral indices was much greater than chance: $z_{SMN} = 7.71, p < 10^{-14}$ and $z_{SMN} = -8.22, p < 10^{-15}$ (Fig. S10H-K).

In general, these results support the hypothesis that global network flexibility is correlated with both the positivity and surprise indices. The correlation with positivity is, in part, mediated by fatigue. These relationships also appear to be driven predominantly, but not exclusively, by flexibility of the somatomotor network.

1.9 Robustness with respect to variation in window length

In the main text we analyzed the time-varying properties of functional brain networks. In order to do so, we estimated the network at different time points by dividing the scan session into 14 equal-sized windows containing 37 samples each. In general, varyinging the number and duration of windows allows us to interrogate the network at different temporal resolutions (*10*); shorter or longer windows facilitate the investigation of correspondingly faster or slower dynamics.

Accordingly, we wished to assess whether variation in these parameters might influence the results presented in the main text. A full exploration of all window sizes is, unfortunately, not practical. Instead, we simply doubled our window length (from 37 TRs to 74 TRs) and halved the number of windows (14 windows to 7 windows). With these new data, we repeated the analysis procedure outlined in the main text. Briefly, this entailed generating time-varying

estimates of functional connectivity, performing community detection, calculating regional and global flexibility, and computing the correlation of those flexibility scores with the positivity and surprise indices.

Interestingly, we observed that the correlation of regional flexibility with both indices was, once again, driven strongly by the somatomotor network (Fig. S11B-F). At the global level, however, flexibility was still strongly correlated with the surprise index ($\hat{r}(SI, F) = -0.35$, $p = 0.0025$) (Fig. S11B) but not correlated with positivity (Fig. S11A) ($\hat{r}(PI, F) = 0.01$, $p = 0.94$).

These results can be interpreted in a number of ways. Principally, it demonstrates the robustness of the relationship between global and regional flexibility with the surprise index. It also suggests that, while robust to other types of variation, the flexibility-positivity correlation is sensitive to the temporal resolution at which we investigate our network.

Nonetheless, these results should be interepreted with some caution. Flexibility (both regional and global) is calculated by averaging changes in community structure over time. With a greater number of time points, these estimates become more stable, i.e. sampling variability becomes small; with fewer time points sampling variability will be proportionally greater. In doubling our window length from 37 TRs to 74 TRs, we halve the number of time points, resulting in a corresponding increase in uncertainty surrounding the flexibility measures and the correlation of those measures with behavioral indices.

In general, there exists a complicated relationship between window size and the uncertainty surrounding network estimation and measurements derived from those networks. With long windows, we get a clearer picture of the network's organization but have few samples with which to characterize its time-varying properties; with shorter windows, the networks we estimate are likely biased by sampling variability (*18*) but may provide more stable estimates of the networks' time varying properites. Mapping out this relationship more precisely, while

important, falls beyond the scope of the present study.

References and Notes

1. T. O. Laumann, *et al.*, *Neuron* **87**, 657 (2015).

2. Y. Benjamini, Y. Hochberg, *Journal of the Royal Statistical Society. Series B (Methodological)* pp. 289–300 (1995).

3. R. A. Poldrack, *et al.*, *Nature Communications* **6** (2015).

4. J. M. Shine, O. Koyejo, R. A. Poldrack, *Proceedings of the National Academy of Sciences* p. www.pnas.org/cgi/doi/10.1073/pnas.1604898113 (2016).

5. P. J. Mucha, T. Richardson, K. Macon, M. A. Porter, J.-P. Onnela, *Science* **328**, 876 (2010).

6. D. S. Bassett, *et al.*, *Chaos: An Interdisciplinary Journal of Nonlinear Science* **23**, 013142 (2013).

7. D. S. Bassett, *et al.*, *Proceedings of the National Academy of Sciences* **108**, 7641 (2011).

8. U. Braun, *et al.*, *Proceedings of the National Academy of Sciences* **112**, 11678 (2015).

9. L. Chai, M. G. Mattar, I. A. Blank, E. Fedorenko, D. S. Bassett, *Cerebral Cortex* **In press** (2016).

10. R. F. Betzel, D. S. Bassett, *NeuroImage* (2016).

11. D. Watson, L. A. Clark, *The PANAS-X: Manual for the positive and negative affect schedule-expanded form* (University of Iowa, 1999).

12. J. D. Power, K. A. Barnes, A. Z. Snyder, B. L. Schlaggar, S. E. Petersen, *Neuroimage* **59**, 2142 (2012).

13. A. J. Zautra, R. Fasman, B. P. Parish, M. C. Davis, *Pain* **128**, 128 (2007).

14. J. R. Shambroom, S. E. Fábregas, J. Johnstone, *Journal of sleep research* **21**, 221 (2012).

15. L. Jeon, J. Finkelstein, *Digital Healthcare Empowering Europeans: Proceedings of MIE2015* **210**, 458 (2015).

16. D. S. Bassett, *et al.*, *PLoS Comput Biol* **9**, e1003171 (2013).

17. Z. Zhang, Q. K. Telesford, C. Giusti, K. O. Lim, D. S. Bassett, *PLoS One* **11**, e0157243 (2016).

18. T. O. Laumann, *et al.*, *Cerebral Cortex* (2016).

active, afraid, alert, alone, amazed, angry, angry-at-self, ashamed, astonished, at-ease, attentive, bashful, blameworthy, blue, bold, calm, cheerful, concentrating, confident, daring, delighted, determined, disgusted, disgusted-with-self, dissatisfied-with-self, distressed, downhearted, drowsy, energetic, enthusiastic, excited, fearless, frightened, guilty, happy, hostile, inspired, interested, irritable, jittery, joyful, lively, loathing, lonely, nervous, proud, relaxed, sad, scared, scornful, shaky, sheepish, shy, sleepy, sluggish, strong, surprised, timid, tired, upset

Table S1: **PANAS-X terms.** Complete list of PANAS-X terms.

PANAS-X Class	PANAS-X Category
**negative affect**	afraid, scared, nervous, jittery, irritable, hostile, guilty, ashamed, upset, distressed
**positive affect**	active, alert, attentive, determined, enthusiastic, excited, inspired, interested, proud, strong
**fear**	afraid, scared, frightened, nervous, jittery, shaky
**hostility**	angry, hostile, irritable, scornful, disgusted, loathing
**guilt**	guilty, ashamed, blameworthy, angry at self, disgusted with self, dissatisfied with self
**sadness**	sad, blue, downhearted, alone, lonely
**joviality**	happy, joyful, delighted, cheerful, excited, enthusiastic, lively, energetic
**self-assurance**	proud, strong, confident, bold, daring, fearless
**attentiveness**	alert, attentive, concentrating, determined
**fatigue**	sleepy, tired, sluggish, drowsy
**serenity**	calm, relaxed, at ease
**surprise**	amazed, surprised, astonished

Table S2: **PANAS-X class assignments.** The first column displays the name of each affect class and the second column displays the PANAS-X scores assigned to that class.

Variable class	Variable name
**N/A**:	subcode, date
**after scan**	anxiety during scan, diastolic, pulse, systolic
**blood**	ba, eo, hgb, ly, mch, mchc, mcv, mo, mpv, ne, plt, rbc, wbc
**date of week**	date of week
**email**	LIWC-CDI, LIWC-negemo, LIWC-posemo
**morning**	pulse, sleep quality, soreness, diastolic, systolic
**previous evening**	alcohol, gut health, how much did tinnitus bother you today, psoriasis severity, stress, time spent outdoors
**rna**	rin
**same evening**	alcohol, gut health, how much did tinnitus bother you today, psoriasis severity, stress, time spent outdoors
**scan**	has breath hold, has dots, has dti, has faceloc, has grid, has n-back, has resting, has superloc, has T1W, has T2W, noise cancel
**weather**	precip, temp hi, temp lo
**weight**	weight
**zeo**	time in deep, time in light, time in REM, total Z, zq

Table S3: **Psychophysiological variables** The first column displays the broad class to which each variable was assigned. The second column lists the specific variable names.

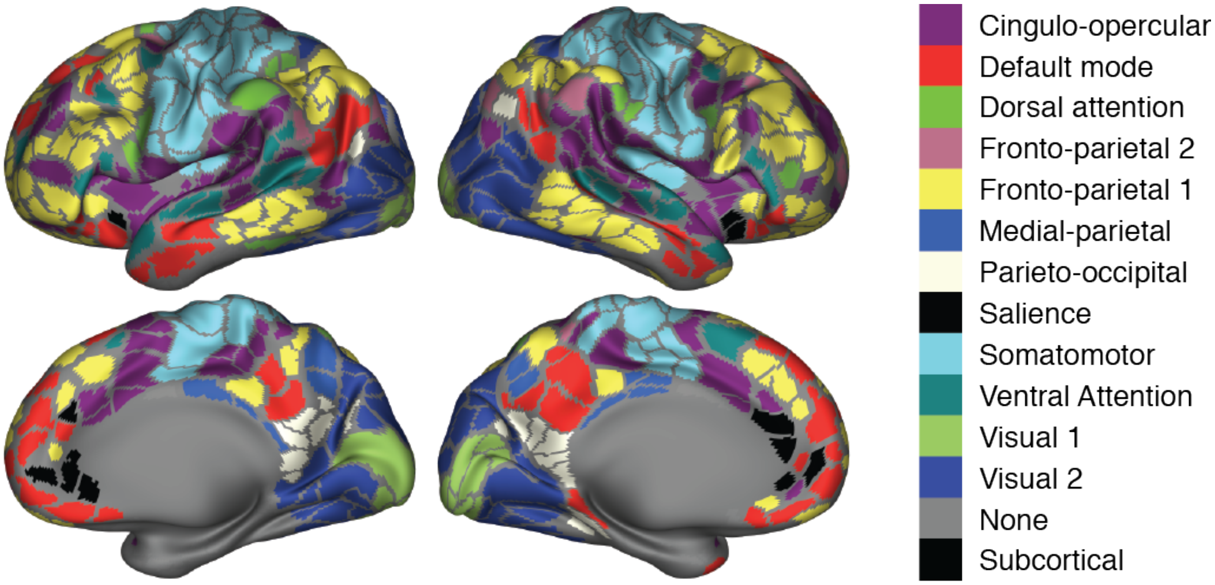

Figure S1: **Cognitive systems.** Topographic distribution of the 12 cortical brain systems (the subcortical system is not shown in this figure).

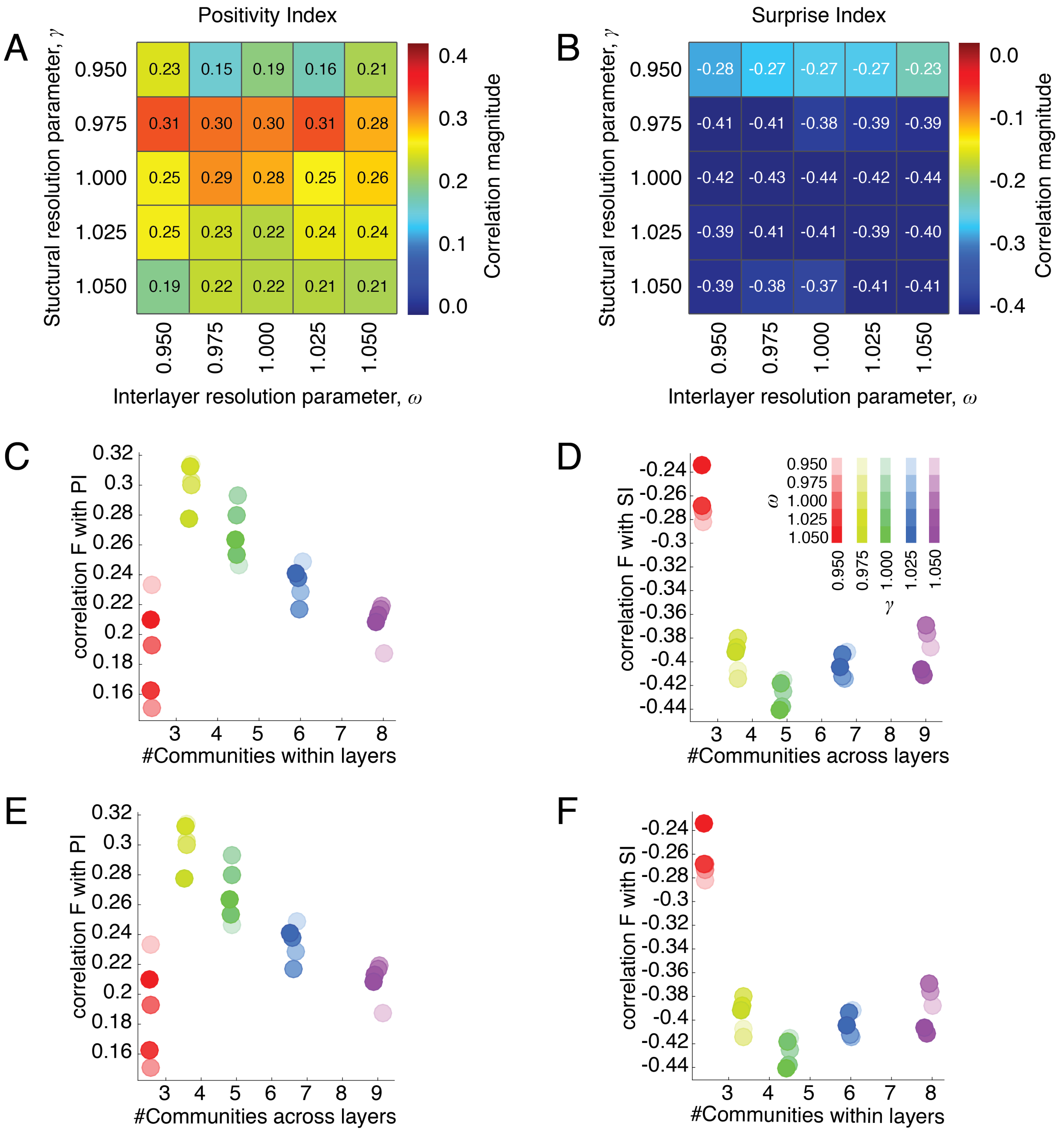

Figure S2: **Variation of resolution parameters.** We varied the resolution parameters, γ and ω, over the range 0.95–1.05 in increments of 0.025. For each pair of parameters, we performed multi-layer community detection as described in the main text. From the detected communities we calculated flexibility scores and the correlation of scores with positivity and suprise indices. Here we show the measured correlation coefficients as a function of γ, ω for the positivity index (A) and the suprise index (B).

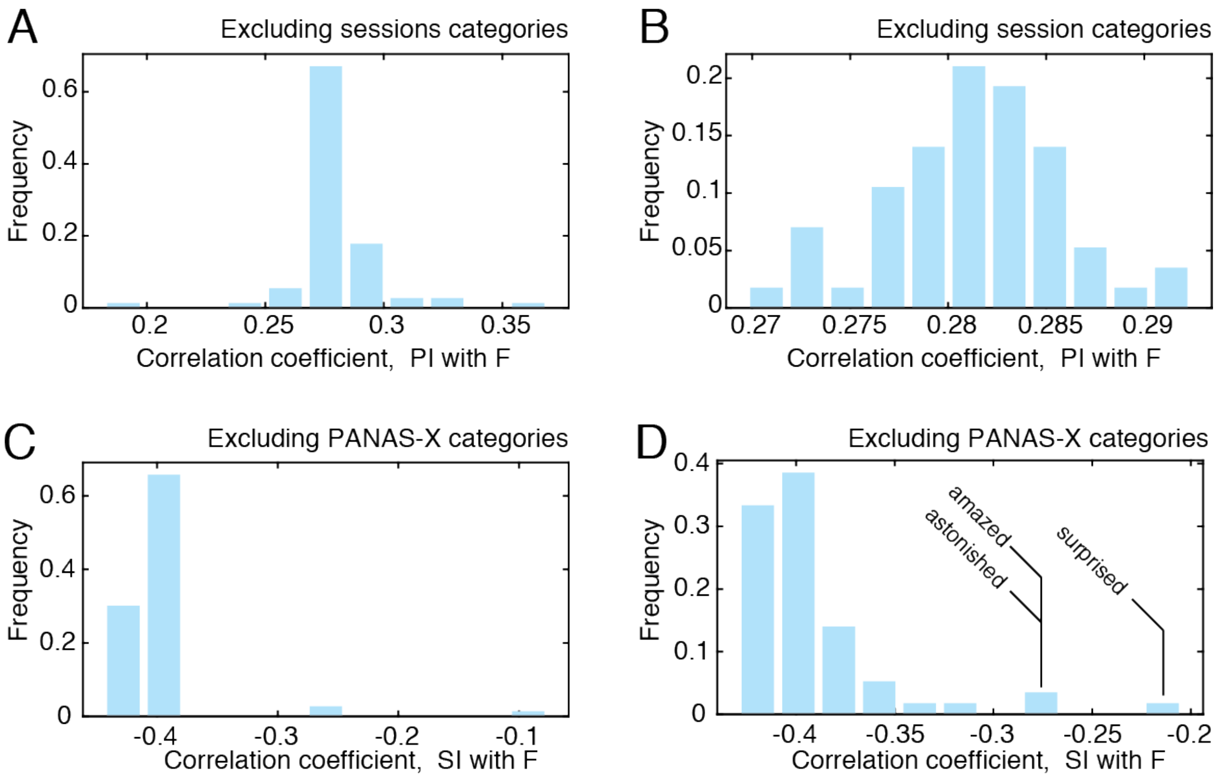

Figure S3: **Results of "Leave one out" analyses.** Correlation coefficients of flexibility with positivity and surprise indices based on limited observations. *(A)* Distribution of correlation coefficients, $\hat{r}(PI, F)$, excluding individual data collection sessions. *(B)* Distribution of correlation coefficients, $\hat{r}(PI, F)$, excluding individual PANAS-X terms. *(C)* Distribution of correlation coefficients, $\hat{r}(SI, F)$, excluding individual data collection sessions. *(D)* Distribution of correlation coefficients, $\hat{r}(SI, F)$, excluding individual PANAS-X terms.

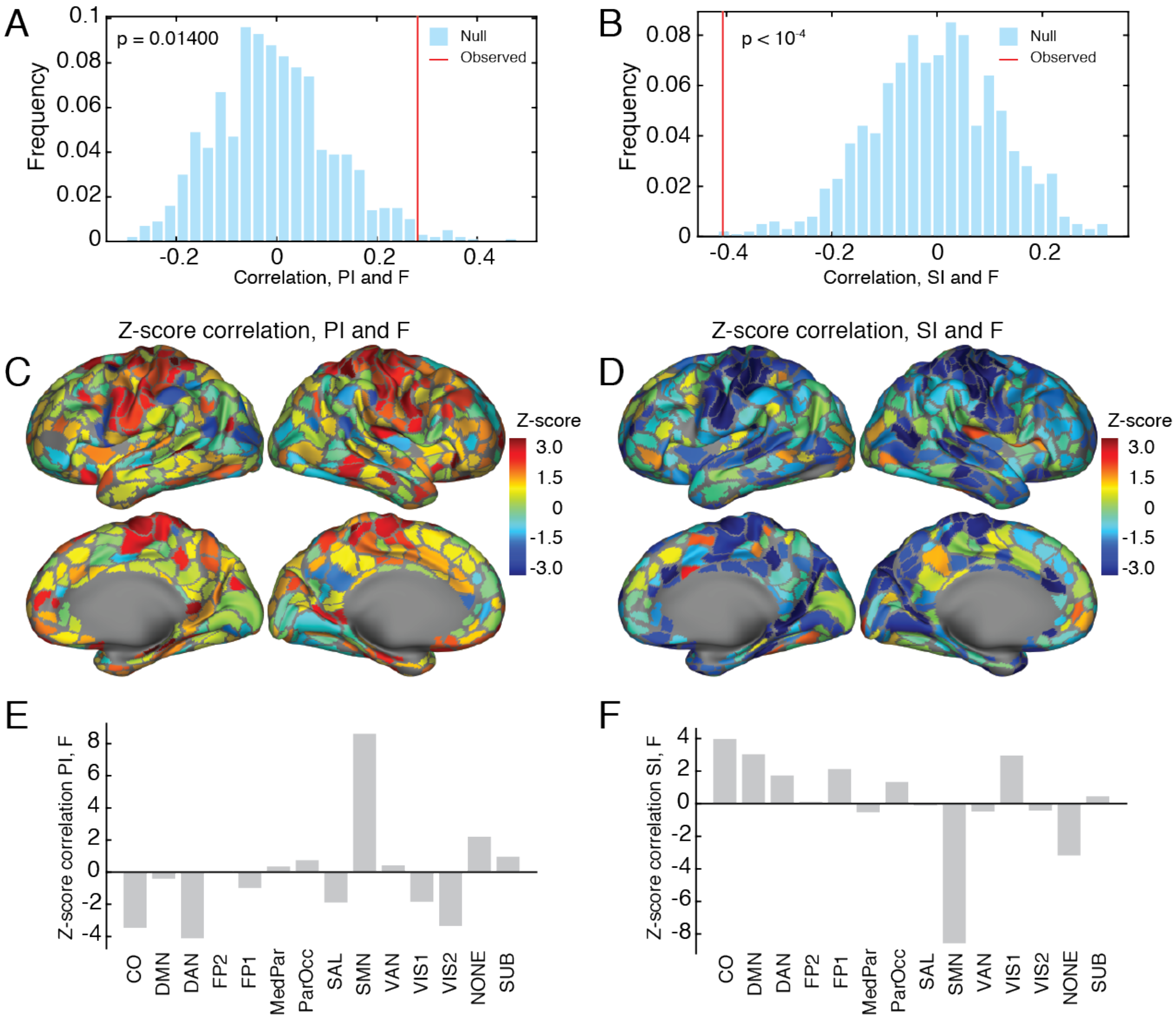

Figure S4: **Results of permutation tests for estimating correlation confidence intervals.** *(A)* Null distribution for the correlation$\hat{r}(PI, F)$. *(B)* Null distribution for the correlation$\hat{r}(SI, F)$. *(C)* Z-scores of correlation PI with regional flexibility. *(D)* Z-scores of correlation SI with regional flexibility. *(E,F)* Region-level correlations from panels *C* and *D* aggregated by cognitive system and expressed as z-scores.

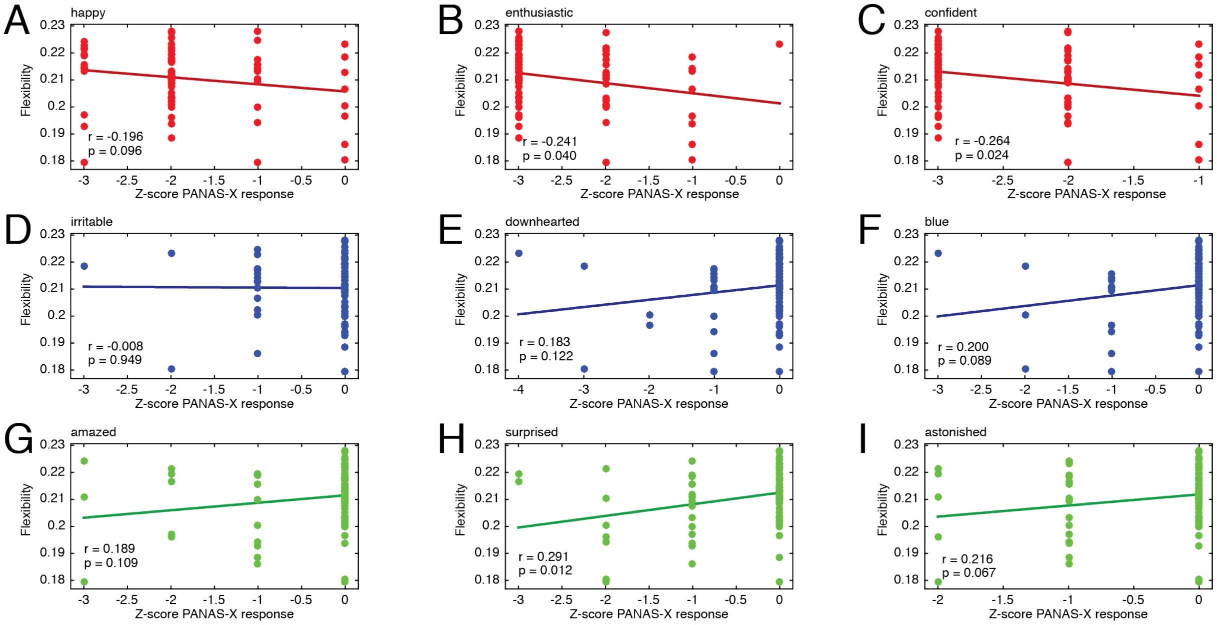

Figure S5: **Individual PANAS-X categories.** *(A,B,C)* Scatterplots of top three PANAS-X components (in terms of their magnitude loading onto PI) versus global flexibility. *(D,E,F)* Scatterplots of bottom three PANAS-X components (in terms of their magnitude loading onto PI) versus global flexibility. *(G,H,I)* Scatterplots of top three PANAS-X components (in terms of their magnitude loading onto SI) versus global flexibility.

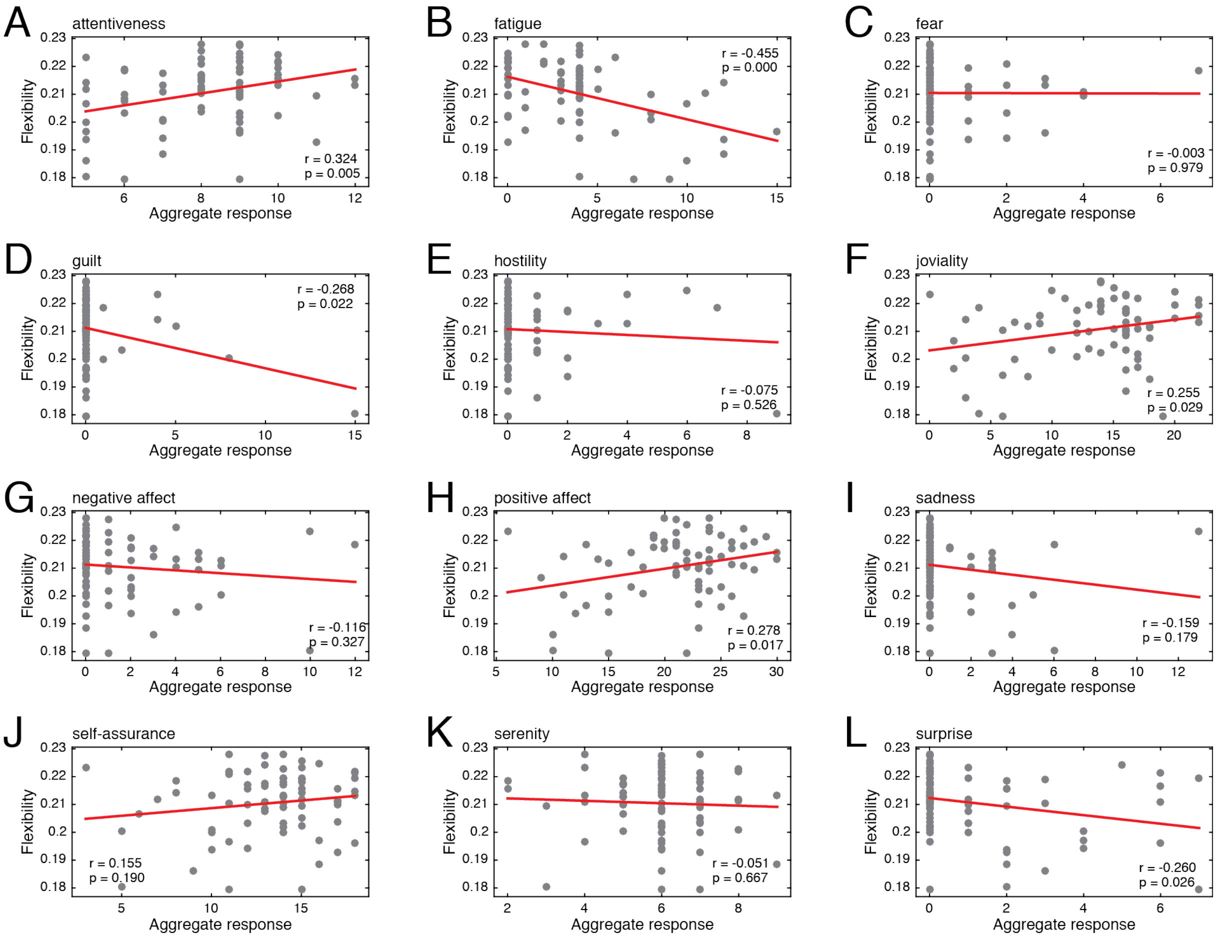

Figure S6: **Correlations of flexibility with affect classes.** Each panel shows the correlation of global flexibility with a different affect class. The aggregate score for each affect class is based on a sum of responses to specific PANAS-X terms.

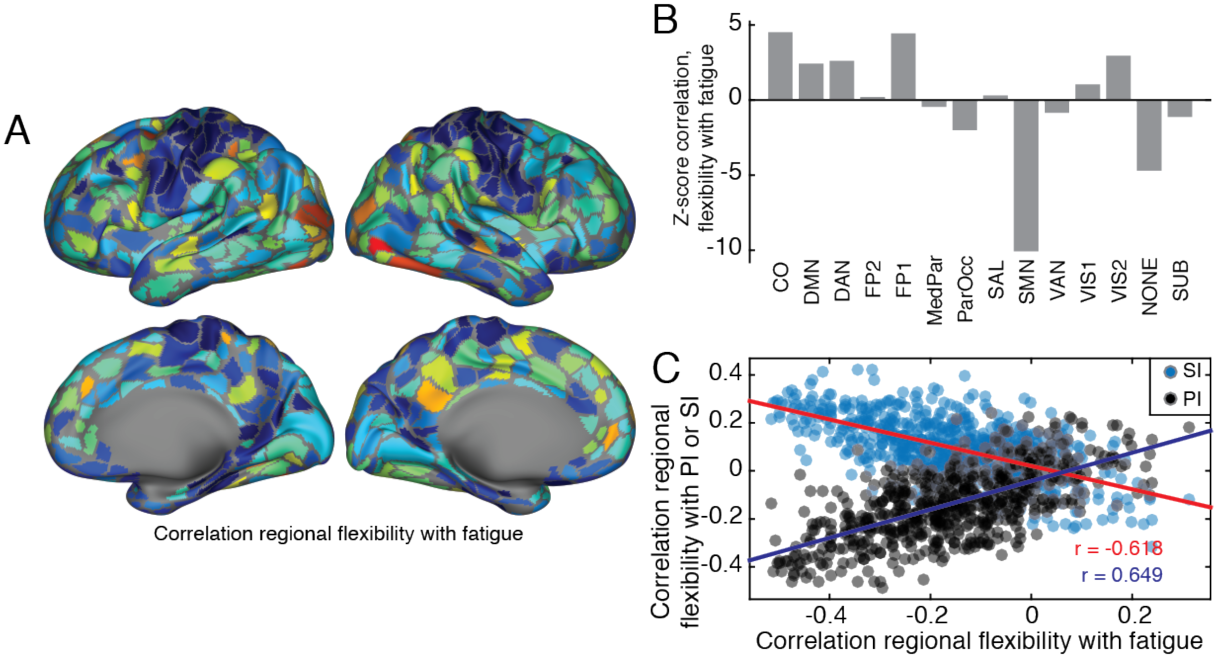

Figure S7: **Correlation of fatigue with flexibility.** *(A)* Topographic distribution of regional flexibility with fatigue. *(B)* Region-level correlations aggregated by cognitive system and expressed as z-scores. (*C*) Scatterplot of $\hat{r}(fatigue, F)$ versus $\hat{r}(PI, F)$ and $\hat{r}(SI, F)$.

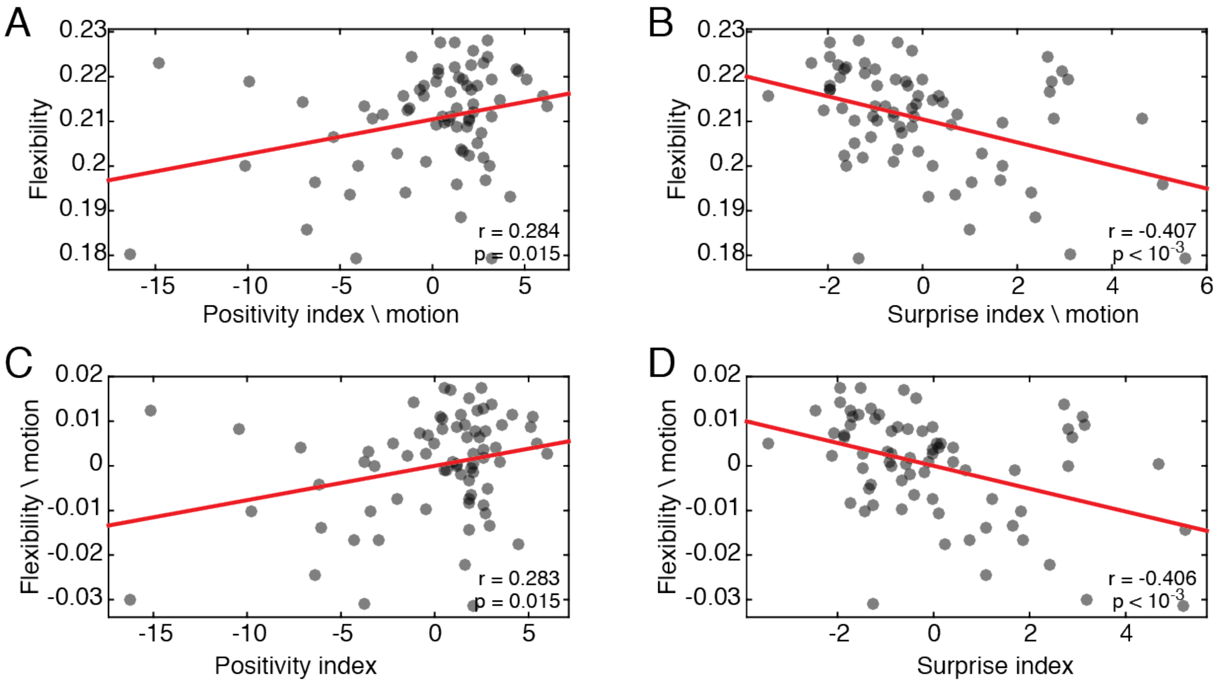

Figure S8: **Accounting for in-scanner head motion.** *(A)* Correlation of positivity index (after regressing out motion) with global flexibility. *(B)* Correlation of surprise index (after regressing out motion) with global flexibility. *(C)* Correlation of positivity index with global flexibility (after regressing out motion). *(D)* Correlation of positivity index with global flexibility (after regressing out motion).

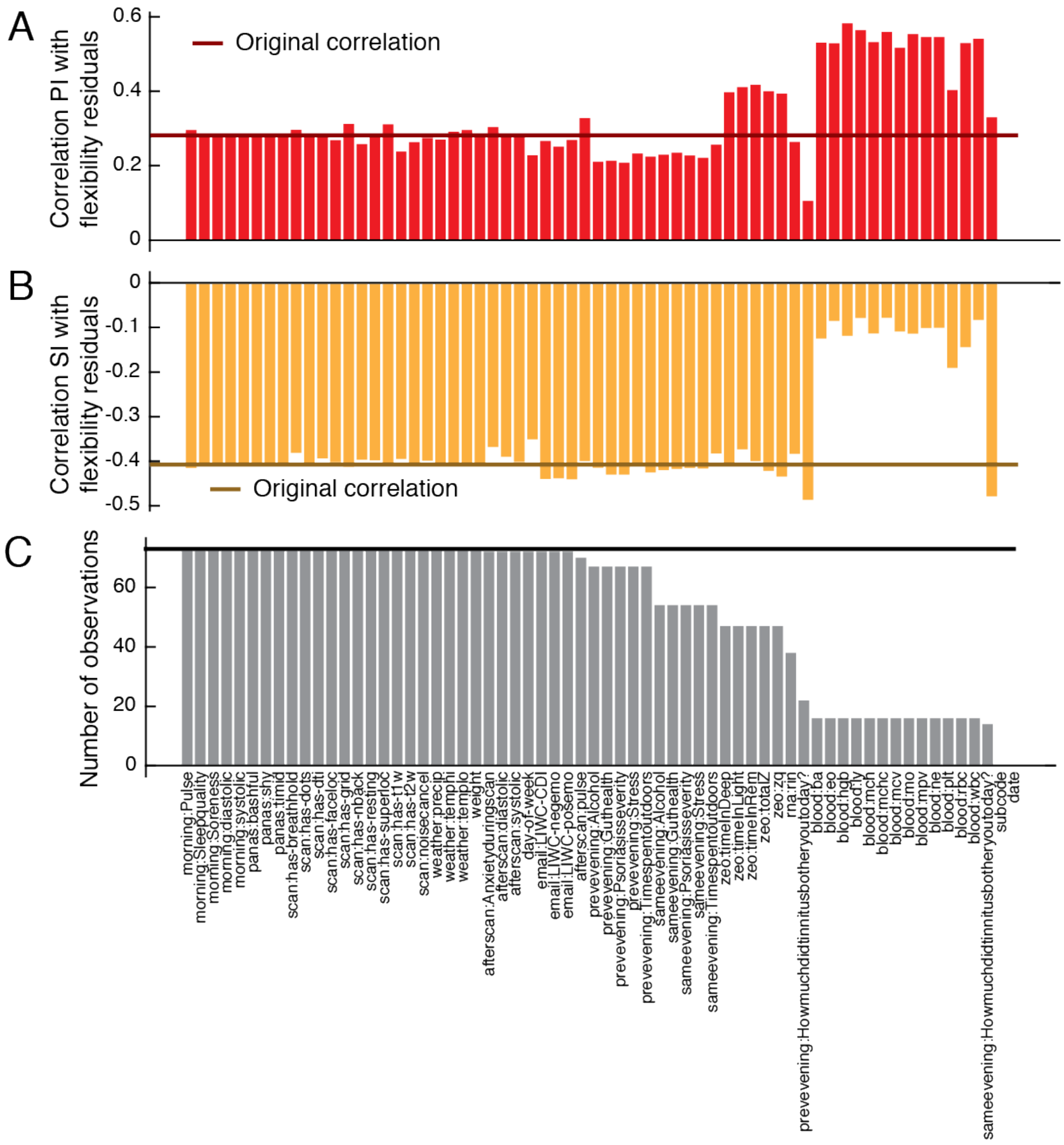

Figure S9: **Accounting for other psychophysiological measurements.** We regressed 64 psychophysiological variables from global flexibility and calculated the correlation of the residuals with both positivity and surprise indices. *(A)* Results after applying these methods to the positivity index. Each bar represents the correlation magnitude of residuals with positivity. The dark red line represents the correlation magnitude without accounting for any psychophysiological measurements. *(B)* Results after applying these methods to the surprise index. Each bar represents the correlation magnitude of residuals with surprise. The dark orange line represents the correlation magnitude without accounting for any psychophysiological measurements. *(C)* The number of data collection sessions for which a given psychophysiological variable was collected.

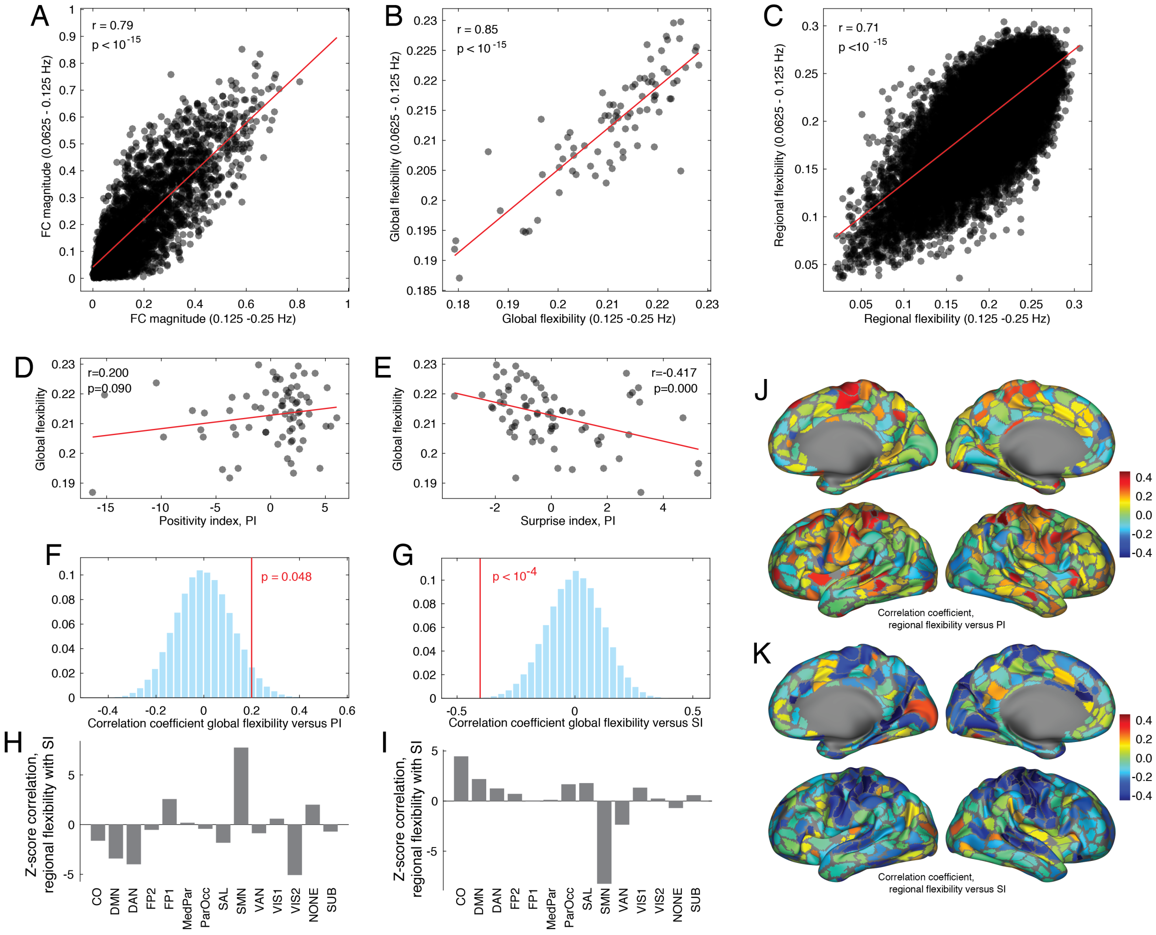

Figure S10: **Corroboration of low-frequency and high-frequency fMRI BOLD signal analysis.** We compared network organization, flexibility metrics, and flexibility/behavior correlations for the high-frequency data described in the main text (12.5 - 25 Hz) and low-frequency data analyzed in a more traditional frequency band (0.0625 - 12.5 Hz). *(A)* Scatter plot depicting the edge-wise relationships of dynamic functional connectivity of low-frequency and high-frequency data (plot depicts data from only one of the 73 scan sessions). *(B)* Scatterplot depicting relationship of *global* flexibility for low- and high-frequency data. *(C)* Scatterplot depicting relationship of *regional* flexibility for low- and high-frequency data. *D* Scatterplot showing correlation of positivity index with global flexibility. *E* Scatterplot showing correlation of surprise index with global flexibility. *F,G* We assessed the statistical significance of these correlations using permutation testing to generate null distribution of correlations; both positivity and surprise indices exhibited statistically significant magnitudes, $p < 0.05$. *H,I* System-level correlations of regional flexibility with positivity and surprise indices. As in the main text, flexibility of the somatomotor network was highly correlated with behavioral indices. *J,K* Topographic distribution of regional flexibility scores with behavioral indices. Again, these plots highlight the contributions of the somatomotor network's flexibility to the overall flexibility/behavior relationships.

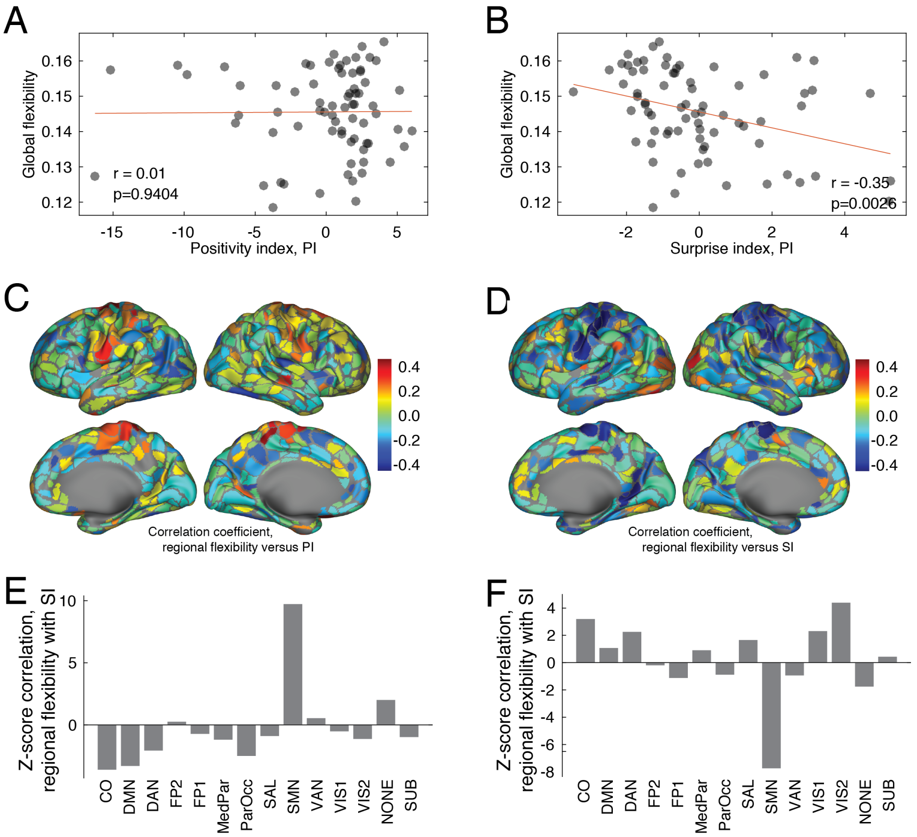

Figure S11: **Effect of changing window size from 37 TRs to 74 TRs.** Scatterplot showing relationship of positivity (*A*) and surprise indices (*B*) with global flexibility calculated from 74 TR windows. We also show the topographic distribution of the correlation of regional flexibility with positivity (*C*) and surprise indices (*D*). As in the main text, regional correlations are strongest in the somatomotor network for both indices (*E*,*F*).
